# LncRNA TUG1 mitigates sepsis-induced acute lung injury via a ceRNA network regulating the CALM1/PRKG1/RYR3/AQP5 axis

**DOI:** 10.1038/s41598-026-51003-1

**Published:** 2026-05-16

**Authors:** Zhe Li, Wan Chen, Lei Shi, Guozheng Qiu, Yao zhou, Yanlin Wei, Zhengzhuang Huang, Liwen Lyu

**Affiliations:** Department of Emergency, The People’s Hospital of Guangxi Zhuang Autonomous Region, Guangxi Academy of Medical Sciences, Nanning, 530021 China

**Keywords:** Sepsis, Acute lung injury, LncRNA TUG1, CeRNA, CALM1, Biomarkers, Diseases, Medical research, Molecular medicine

## Abstract

Sepsis-induced acute lung injury (ALI) is a life-threatening condition associated with high mortality, yet the molecular mechanisms driving alveolar damage remain incompletely understood. Long non-coding RNA (lncRNA) TUG1 has been implicated in organ injury, but its specific role and regulatory network in septic ALI have not been fully elucidated.Using lipopolysaccharide (LPS)-treated mouse lung epithelial (MLE-12) cells and a murine model of ALI, we investigated the functional role of TUG1 through overexpression strategies. Gene expression, protein levels, inflammatory cytokines, and miRNA interactions were assessed via qPCR, Western blotting, ELISA, and dual-luciferase reporter assays. Bioinformatic analysis of public datasets (GSE241238, GSE48080) was performed to validate clinical relevance.TUG1 expression was significantly downregulated in LPS-induced ALI models. TUG1 overexpression mitigated inflammation and oxidative stress by acting as a competing endogenous RNA (ceRNA) for miR-222-3p, thereby derepressing CALM1. Activation of CALM1 subsequently engaged the PRKG1/RYR3 signaling cascade, leading to restoration of AQP5-mediated alveolar fluid clearance. Analysis of public datasets confirmed suppression of the TUG1/CALM1 axis in septic patients and revealed its association with adverse survival outcomes. While the preliminary sample sizes (*n* = 3 in vitro, *n* = 5 in vivo) limit the statistical power (post-hoc 0.65), the robust trends support the TUG1 axis as a potential target, though larger cohort validation is needed.This study identifies TUG1 as a potential modulator of sepsis-induced ALI through the miR-222-3p/CALM1/PRKG1/RYR3/AQP5 axis. These findings underscore the therapeutic potential of targeting TUG1 to alleviate septic lung injury. These findings identify TUG1 as a protective lncRNA that acts via the miR-222-3p/CALM1 axis to regulate calcium signaling and alveolar fluid clearance. Targeting this pathway may offer a novel therapeutic strategy for sepsis-induced ALI.

**Supplementary Information:**

The online version contains supplementary material available at 10.1038/s41598-026-51003-1.

## Introduction

Sepsis-induced acute lung injury (ALI) remains a formidable challenge in critical care medicine, accounting for over 40% of sepsis-related mortality due to persistent alveolar-capillary barrier disruption and excessive inflammation^1–3^. Despite advances in ventilatory support and immunomodulatory therapies, clinical outcomes remain poor, with fewer than 30% of patients achieving functional lung recovery within 28 days^4,5^. The failure of existing therapies highlights an urgent need to elucidate the molecular mechanisms governing alveolar epithelial homeostasis and repair during septic insult.

Recent breakthroughs in transcriptomics and high-throughput sequencing have shed light on the pivotal roles of long non-coding RNAs (lncRNAs) in regulating inflammatory and oxidative responses^6–8^. These non-coding transcripts, typically over 200 nucleotides in length, function through diverse mechanisms—including acting as scaffolds, molecular decoys, or most notably, as competing endogenous RNAs (ceRNAs) that sponge microRNAs (miRNAs) to modulate downstream gene expression^9–11^. Within the context of sepsis and ALI, several lncRNAs have emerged as crucial modulators: NEAT1, for instance, promotes neutrophil extracellular trap (NET) formation via miR-150-5p/HMGB1 signaling^12^, while MALAT1 mitigates endothelial dysfunction by targeting the miR-26a-5p/TET1 axis^13^.

Among these, taurine-upregulated gene 1 (TUG1) has gained attention due to its context-specific effects across different organs in sepsis. While studies have reported a protective role of TUG1 in septic renal and intestinal injury via modulation of apoptosis and autophagy-related pathways^14–16^, contrasting evidence suggests that TUG1 may also aggravate systemic inflammation by enhancing macrophage activation^17^. These findings underscore the tissue-specific complexity of lncRNA-mediated regulation and prompt further investigation of TUG1’s role in sepsis-induced lung injury, particularly in alveolar epithelial cells (AECs)—the primary targets in ALI.

Our preliminary transcriptomic analyses revealed a marked downregulation of TUG1 in lung tissues from sepsis-associated ALI patients, inversely correlating with disease severity (unpublished data). Bioinformatic predictions further identified a conserved ceRNA network wherein TUG1 sponges miR-222-3p, a microRNA implicated in inflammatory amplification and calcium dysregulation^18,19^. This interaction may derepress calmodulin 1 (CALM1), a key calcium sensor involved in regulating the PRKG1/RYR3 signaling cascade—critical for maintaining aquaporin-5 (AQP5)-mediated alveolar fluid clearance^20,21^. However, whether TUG1 modulates this axis to exert a protective role in ALI remains unexplored.

In this study, we delineate the functional significance of the TUG1/miR-222-3p/CALM1 axis in the pathogenesis of LPS-induced ALI. Through in vitro and in vivo models, we demonstrate that TUG1 overexpression alleviates inflammation and oxidative stress by targeting miR-222-3p and restoring CALM1-PRKG1-RYR3 signaling, thereby enhancing AQP5-mediated alveolar fluid transport^22^. Furthermore, analysis of independent clinical cohorts (GSE241238 and GSE48080)^23^ confirms that repression of this axis is associated with poor survival, while its restoration predicts favorable outcomes. These findings not only clarify the lung-specific function of TUG1 but also establish a novel regulatory axis with therapeutic potential in sepsis-associated ALI.

## Materials and methods

### Cell Culture and Transfection

MLE-12 murine alveolar epithelial cells (CRL-2116, ATCC) were propagated in DMEM/F12 (Gibco). Culture media were enriched with 10% heat-inactivated FBS (Gibco), penicillin (100 units/mL), and streptomycin (100 µg/mL) from Beyotime. Standard incubation conditions (37 °C, 5% CO₂, humidified atmosphere) were maintained throughout.

Genetic modifications were conducted using:

- Plasmid transfection: 2 µg/mL pcDNA3.1-TUG1 construct or empty vector (OE-NC) delivered via Lipofectamine 3000 (Invitrogen).
- miRNA regulation: RNAiMAX-mediated transfection (Invitrogen) of miR-222-3p mimic (50 nM), inhibitor (100 nM), or matched controls.

### Metabolic Activity Profiling

Cellular proliferation capacity was evaluated at 24, 48, and 72 h after genetic manipulation. MLE-12 cells (2 × 10³ cells/well in 96-well format) were incubated with CCK-8 solution (Dojindo) for 2 h, followed by optical density measurement at 450 nm using SpectraMax iD5 (Molecular Devices).

### Transcriptional Profiling

Total RNA isolation was performed with TRIzol (TIANGEN), followed by reverse transcription using PrimeScript RT Master Mix (Solarbio) for mRNA/lncRNA or RevertAid RT Kit (Thermo Fisher) for miRNA. Specifically, for miRNA quantification, a standard poly(A) tailing methodology was employed. Total RNA was first polyadenylated and subsequently reverse-transcribed using a universal adapter primer to generate cDNA. Quantitative amplification was carried out on QuantStudio 5 (Applied Biosystems) with SYBR Green chemistry (Vazyme). The “Universal reverse primer” used for miRNA and snoRNA202 amplification was provided as a proprietary component within the commercial miRNA qPCR detection kit, and it specifically anneals to the universal adapter sequence introduced during the reverse transcription step. Data normalization employed GAPDH (cellular transcripts) and snoRNA202 (miRNAs), with relative quantification using 2^(−ΔΔCt) methodology. Primer sequences:

**Table:** 

Gene	Forward (5’→3’)	Reverse (5’→3’)
TUG1	TGAAGCCCCCATTTGAGTCC	CACCCTTCAGGCACCCTATG
CALM1	GTGTCCTCAGCCACCTTCAGAC	ATTCAGCAATCTGCTCTTCAGTCAG
AQP5	CTCCCTAGCATCCTCTCAGC	CACACCCAAGTGTCCCATCA
GAPDH	CAGCCTTCCTTCTTGGGTAT	TGGCATAGAGGTCTTTACGG
miR-222-3p	AGCUACAUCUGGCUACUGGGU	Universal reverse primer
snoRNA202	CTCGCTTCGGCAGCACA	AACGCTTCACGAATTTGCGT

* Note: The Universal reverse primer is a proprietary component provided within the commercial miRNA qPCR kit, designed to bind the universal poly(A) adapter.

### Luciferase Reporter Assay

Luciferase reporter constructs containing wild-type or mutant 3′UTR sequences regions of TUG1 and CALM1 were generated in pmirGLO backbone (Promega). HEK293T cells received dual transfection of reporter plasmids (200 ng) with miR-222-3p mimics/controls. Luminescent signals were quantified 48 h later using Dual-Glo System (Promega), and normalized to Renilla luciferase activity to control for transfection efficiency and cell viability.

### Cytokine Quantification by ELISA

Cytokine detection: Secreted inflammatory mediators (TNF-α, IL-6, IL-1β) were quantified in biological samples using ELISA kits (Bio-Swamp) with Synergy H1 microplate reader (BioTek) detection at 450 nm.

### Western Blotting

Protein lysates were prepared with RIPA buffer (Beyotime), quantified via BCA assay (Solarbio), and resolved on 10% SDS-PAGE gels. After transfer to PVDF membranes (Millipore), blots were probed with antibodies against CALM1 (ab45689, 1:1,000), PRKG1 (#13511, CST, 1:1,000), RYR3 (ab2868, 1:1,000), AQP5 (ab78486, 1:1,000; Abcam), and β-actin (AC026, ABclonal, 1:2,000). HRP-conjugated secondary antibodies (ABclonal, 1:5,000) and chemiluminescence detection (Millipore) were used for visualization. Band intensities were quantified using ImageJ.

### Animal Experiments

All procedures complied with ARRIVE guidelines and were approved by the Institutional Ethics Committee (KY-5Y-2023-014). C57BL/6 male mice (20–22 g, Guangxi Medical University) were acclimated in SPF facilities with free access to feed and water. Experimental groups included:

- Sham group: Received intraperitoneal (i.p.) injection of phosphate-buffered saline (PBS).
- LPS: i.p. injection of 10 mg/kg LPS (E. coli O111:B4, Sigma).
- LPS + OE-TUG1: Briefly, mice were anesthetized, and an intratracheal administration of AAV9-TUG1 (1 × 10¹¹ vg/mouse, HanBio) suspended in 50 µL of sterile PBS was performed exactly 7 days prior to the i.p. LPS injection to ensure optimal viral transduction and robust lung-specific gene expression.
- LPS + AAV-NC: Mice received an intratracheal injection of empty AAV9 (1 × 10¹¹ vg/mouse in 50 µL PBS) 7 days prior to the LPS challenge.

Animals were sacrificed 24 h after LPS treatment for tissue collection. At the end of the experiment, mice were euthanized by intraperitoneal injection of sodium pentobarbital (100 mg/kg). After loss of reflexes, cervical dislocation was performed to ensure complete euthanasia.

### Histopathology and Lung Edema Assessment

Lung specimens were fixed in 4% PFA, embedded in paraffin, and sectioned at 5 μm thickness. H&E-stained sections (Solarbio) were imaged under Olympus BX53 for blinded histopathological scoring (0–4 scale) based on characteristic injury markers. Pulmonary edema was quantified via wet/dry weight ratio determination after 72 h desiccation at 65 °C.

### Bioinformatics Analysis

Target Prediction and Selection: The competing endogenous RNA (ceRNA) network was constructed based on in silico predictions. Specifically, the StarBase database (ENCORI, https://starbase.sysu.edu.cn) was utilized to screen for miRNAs interacting with TUG1, with filters set for high stringency (CLIP-Data ≥ 2). To identify downstream mRNA targets of miR-222-3p, the TargetScan database (Release 8.0, https://www.targetscan.org) was employed. Candidate genes were subsequently filtered based on their biological relevance to calcium signaling and alveolar fluid clearance, leading to the selection of CALM1.

Public datasets GSE241238 (PBMCs: 4 sepsis vs. 4 controls) and GSE48080 (longitudinal profiles: 5 survivors vs. 5 non-survivors) were analyzed using R (v4.2.1). Differential expression was calculated using DESeq2 with thresholds of |log₂FC| > 1 and FDR < 0.05. Co-expression networks were built using WGCNA, and survival relevance was evaluated with the limma package.

### Statistical Analysis

Continuous variables presented as mean ± SD. Multiple testing was corrected using the False Discovery Rate (FDR) < 0.05, with one-way ANOVA applied after Shapiro-Wilk normality testing for comparisons among ≥ 3 groups. Unpaired Student’s t-test was used for dual-group comparisons. Nonparametric datasets were evaluated using the Mann-Whitney U test. Furthermore, post-hoc power calculations indicated a statistical power of approximately 0.65 for our current sample sizes (in vitro n = 3, in vivo n = 5). While this power is sufficient to identify the significant main effects observed in this preliminary study, we acknowledge that larger cohorts will be required for future extensive validations. ’’Correlation analyses utilized Pearson’s coefficient. Significance threshold was set at *P* < 0.05. All computations were executed in GraphPad Prism 9.0; partial figure processing was conducted using R (version 4.4.3)Illustrative pathway and schematic figures were created using BioRender (https://biorender.com).

## Result

### TUG1 overexpression alleviates LPS-induced lung epithelial cell injury

**Fig. 1:**
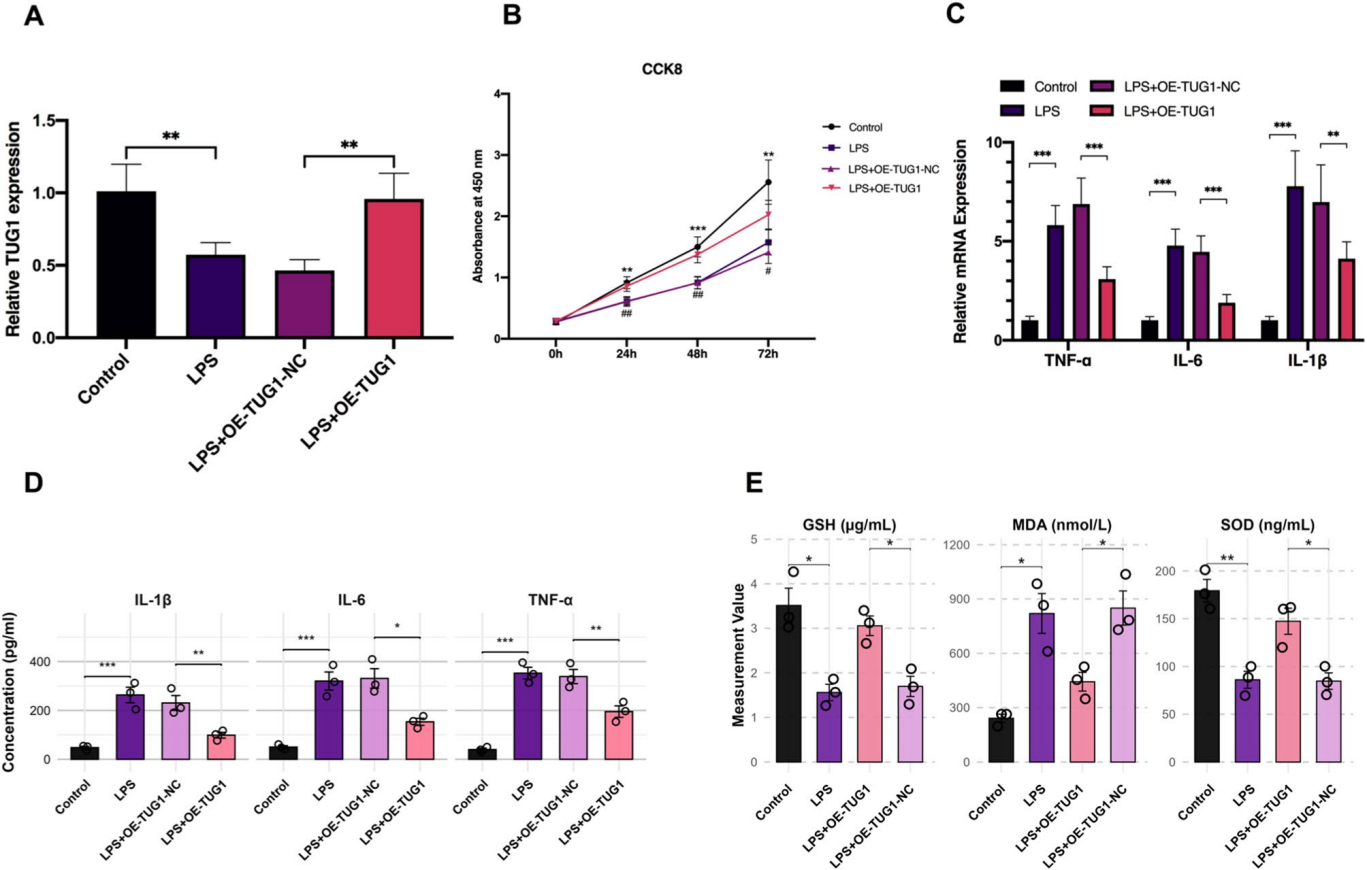
TUG1 overexpression attenuates LPS-induced injury in MLE-12 cells **(A)** Quantitative PCR (qPCR) analysis of TUG1 mRNA expression in control, LPS-treated, and TUG1-overexpressing MLE-12 cells. LPS markedly reduced TUG1 levels, which were restored by TUG1 overexpression. **(B)** Cell viability assessed by CCK-8 assay. LPS significantly decreased cell viability, while TUG1 overexpression partially rescued this effect. **(C)** qPCR analysis of pro-inflammatory cytokine mRNA levels (TNF-α, IL-6, IL-1β). LPS elevated cytokine expression, which was suppressed by TUG1 overexpression. **(D)** ELISA measurement of secreted TNF-α, IL-6, and IL-1β in cell supernatants. TUG1 overexpression reduced LPS-induced cytokine secretion. **(E)** Oxidative stress markers evaluated by ELISA: GSH and SOD activities were decreased, and MDA content increased upon LPS treatment; these changes were reversed by TUG1 overexpression. *Data are presented as mean ± SD, *n* = 3. *P < 0.05, **P < 0.01, ***P < 0.001 vs. control; #*P* < 0.05, ##*P* < 0.01 vs. LPS + NC (one-way ANOVA with Tukey’s test).

To explore the role of TUG1 in acute lung injury (ALI), we established an in vitro model by treating mouse lung epithelial cells (MLE-12) with LPS (1 µg/mL, 24 h). TUG1 expression was significantly downregulated in LPS-treated cells compared to untreated controls, while transfection with a TUG1 overexpression plasmid effectively restored its expression (Fig. 1A). LPS exposure markedly reduced cell viability, as evidenced by CCK-8 assays. However, overexpression of TUG1 significantly attenuated this cytotoxic effect (Fig. 1B). We further investigated the impact of TUG1 on inflammatory responses. LPS stimulation upregulated mRNA levels of TNF-α, IL-6, and IL-1β, and these increases were mirrored in the secretory levels of these cytokines in cell supernatants (Fig. 1C-D). TUG1 overexpression suppressed both mRNA expression (rather than direct transcriptional activity, as ChIP was not performed) and secretory hyperactivation of inflammatory mediators (Fig. 1C-D). To assess oxidative stress, we measured glutathione (GSH), superoxide dismutase (SOD), and malondialdehyde (MDA). LPS treatment reduced GSH and SOD activities while elevating MDA content, indicating oxidative damage. TUG1 overexpression reversed these perturbations, restoring antioxidant capacity and reducing lipid peroxidation (Fig. 1E). These results demonstrate that TUG1 protects lung epithelial cells against LPS-induced injury by mitigating inflammation and oxidative stress.

### TUG1 attenuates LPS-induced injury by sponging miR-222-3p

**Fig. 2:**
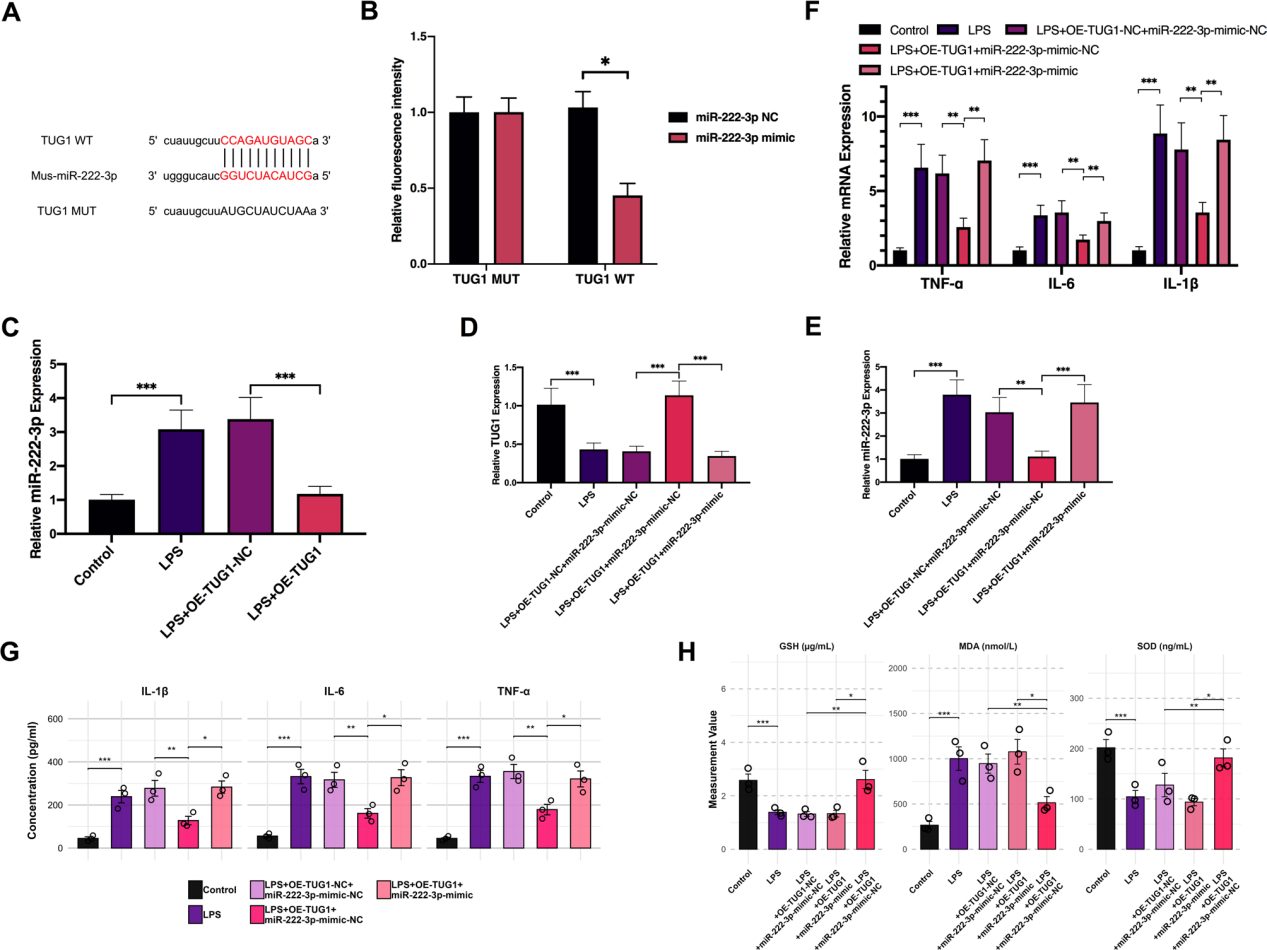
TUG1 mitigates LPS-induced injury by targeting miR-222-3p **(A)** Predicted binding site (purple) between TUG1 (red) and miR-222-3p (green) identified via StarBase. **(B)** Dual-luciferase assay in HEK293T cells co-transfected with miR-222-3p mimic and wild-type (TUG1-WT) or mutant (TUG1-MUT) reporters. miR-222-3p mimic significantly reduced luciferase activity in the TUG1-WT group but not in TUG1-MUT. **(C)** qPCR of miR-222-3p in MLE-12 cells treated with LPS and/or TUG1 overexpression. LPS increased miR-222-3p expression, which was suppressed by TUG1. **(D–E)** qPCR analysis of TUG1 (D) and miR-222-3p (E) in cells co-transfected with TUG1 plasmid and miR-222-3p mimic. miR-222-3p mimic diminished TUG1 overexpression efficiency and restored miR-222-3p levels. **(F)** qPCR quantification of TNF-α, IL-6, and IL-1β. TUG1 suppressed LPS-induced cytokine transcription, which was reversed by miR-222-3p mimic. **(G)** ELISA detection of cytokine secretion. TUG1 reduced inflammatory cytokine release, which was counteracted by miR-222-3p mimic. **(H)** Oxidative stress markers (ELISA). TUG1 reversed LPS-induced GSH/SOD reduction and MDA elevation; these effects were abolished by miR-222-3p mimic. *Data represent mean ± SD of three independent experiments. One-way ANOVA: *P < 0.05, **P < 0.01, ***P < 0.001 vs. control; #*P* < 0.05, ##*P* < 0.01 vs. LPS + NC.

To delineate the mechanism of TUG1, we first performed bioinformatics analysis using the StarBase database to screen for potential miRNA targets of TUG1. Among the predicted candidates, miR-222-3p was prioritized based on its established role in inflammatory regulation and its high binding score in the StarBase algorithm. StarBase predicted a specific complementary binding site within the TUG1 sequence (nucleotides 1456–1472) (Fig. 2A). To validate this interaction, dual-luciferase reporter assays were conducted. The miR-222-3p mimic significantly reduced the luciferase activity of wild-type TUG1 (TUG1-WT) by approximately 1.5-fold (*P < 0.001), whereas mutation of the predicted seed sequence abolished this inhibitory effect in the mutant construct (TUG1-MUT) (Fig. 2B). These findings support a direct interaction between TUG1 and miR-222-3p. Although RNA immunoprecipitation (RIP) assays would further strengthen the evidence of direct binding, the reporter data provide functional validation of the predicted targeting relationship.

We next examined the reciprocal regulatory relationship between TUG1 and miR-222-3p. LPS treatment significantly upregulated miR-222-3p expression, whereas TUG1 overexpression suppressed miR-222-3p levels (*P < 0.05, Fig. 2C). Conversely, co-transfection of miR-222-3p mimic with the TUG1 overexpression plasmid reversed the inhibitory effect of TUG1 on miR-222-3p and led to a reduction in TUG1 expression (*P < 0.05, Fig. 2D-E), indicating a potential negative regulatory feedback loop.

To further determine the functional relevance of this interaction, we modulated miR-222-3p expression in TUG1-overexpressing MLE-12 cells. TUG1 overexpression significantly attenuated LPS-induced upregulation of pro-inflammatory cytokines TNF-α, IL-6, and IL-1β *(qPCR showed reduced cytokine mRNA levels, which were significantly reversed by miR-222-3p mimic; Fig. 2F, *P* < 0.01) and protein levels as determined by ELISA/Western blot analysis (Fig. 2G). Importantly, co-transfection with miR-222-3p mimic largely abolished these anti-inflammatory effects, suggesting that miR-222-3p mediates, at least in part, the protective role of TUG1. Similarly, TUG1 overexpression restored antioxidant capacity, as evidenced by increased GSH and SOD activities and reduced MDA accumulation following LPS stimulation. These effects were significantly reversed by miR-222-3p mimic (*P < 0.05, Fig. 2H), indicating that TUG1 modulates oxidative stress responses through miR-222-3p.

Collectively, these results indicate that TUG1 exerts protective effects against LPS-induced inflammatory and oxidative injury by functionally interacting with and suppressing miR-222-3p, thereby supporting the existence of a TUG1/miR-222-3p regulatory axis in sepsis-related acute lung injury.

### TUG1 acts as a ceRNA to relieve miR-222-3p-mediated repression of CALM1

**Fig. 3:**
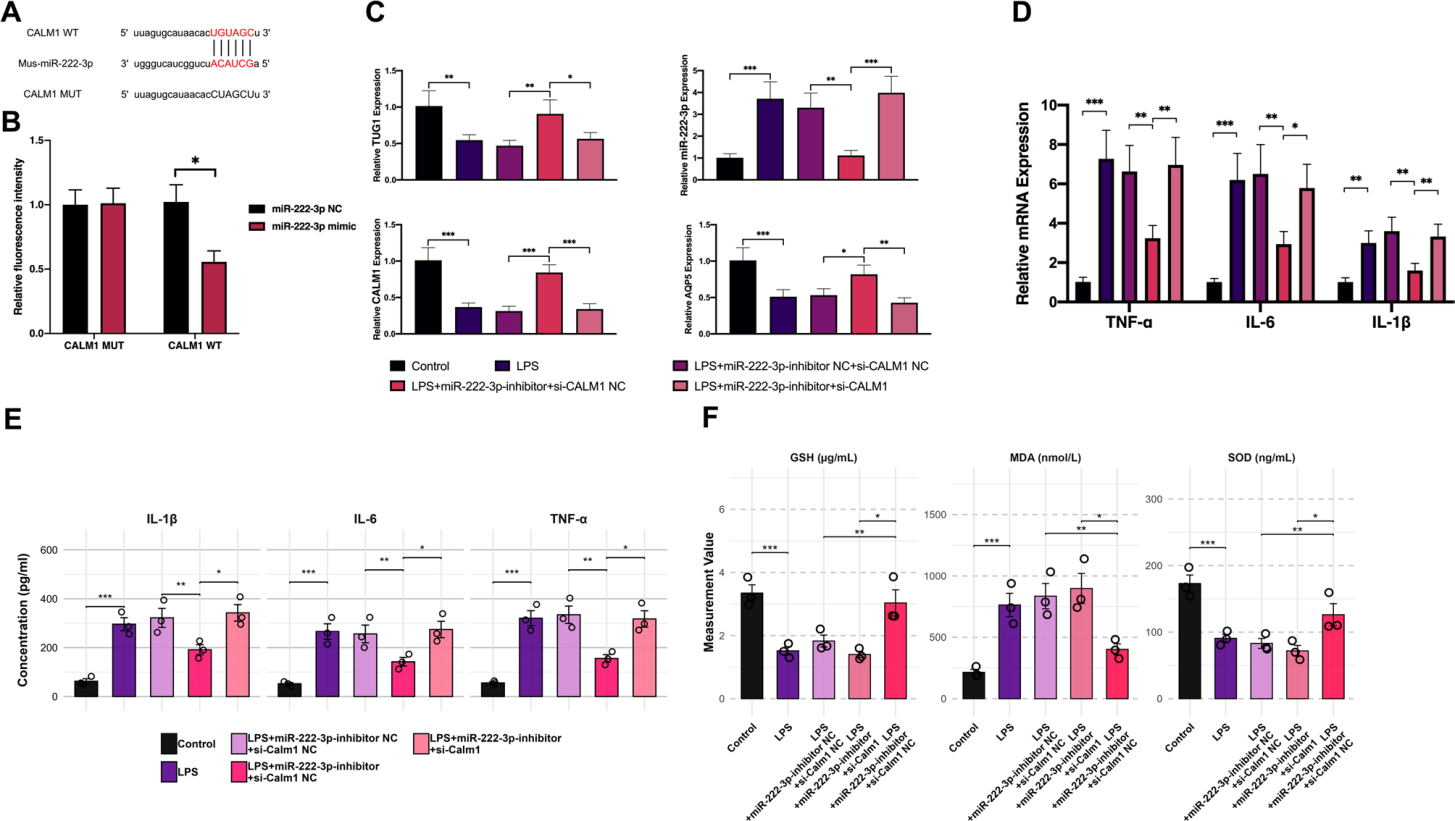

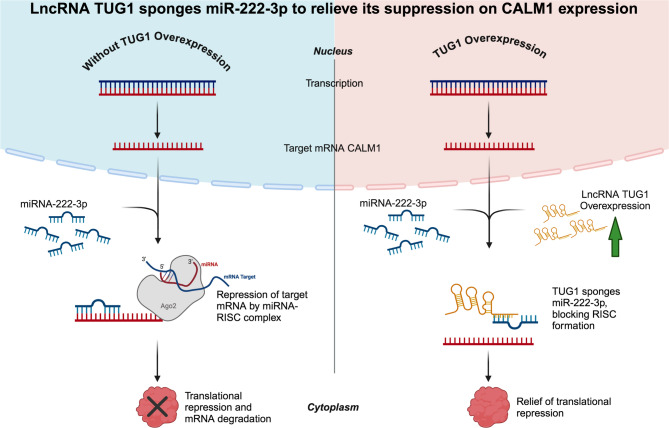
TUG1 functions as a ceRNA to derepress CALM1 by sequestering miR-222-3p **(A)** Predicted miR-222-3p binding site within the 3′UTR of CALM1, as identified by TargetScan. **(B)** Dual-luciferase assay showing decreased luciferase activity in CALM1-WT but not CALM1-MUT when co-transfected with miR-222-3p mimic. **(C)** qPCR analysis of TUG1, miR-222-3p, CALM1, and AQP5 following miR-222-3p inhibition or CALM1 silencing. **(D–E)** mRNA (qPCR) and protein (ELISA) levels of TNF-α, IL-6, and IL-1β. **(F)** Oxidative stress biomarkers (ELISA): GSH and SOD activities, and MDA content. **(G)** Schematic illustration of the TUG1/miR-222-3p/CALM1 regulatory axis, where TUG1 acts as a miRNA sponge, preventing miR-222-3p from targeting CALM1, thus enabling its translation. The schematic model in panel (G) was created with BioRender. *Data are shown as mean ± SD, *n* = 3. *P < 0.05, **P < 0.01, ***P < 0.001 vs. control; #*P* < 0.05, ##*P* < 0.01 vs. LPS + NC (one-way ANOVA with Tukey’s test).

To dissect the downstream effectors of the TUG1/miR-222-3p axis, we utilized TargetScan to predict mRNAs targeted by miR-222-3p. By filtering the candidates for genes intrinsically involved in calcium homeostasis—a critical pathway for alveolar fluid clearance—CALM1 emerged as a high-scoring target, with a conserved miR-222-3p binding site predicted in its 3’UTR (Fig. 3A). Dual-luciferase reporter assays validated this interaction: miR-222-3p mimic significantly inhibited the luciferase activity of wild-type CALM1 (CALM1-WT) by approximately 0.65-fold (95% CI 0.55–0.75, Cohen’s d = 1.8), but not the mutant (CALM1-MUT) (*P* < 0.05, Fig. 3B), confirming direct targeting. We further explored the reciprocal regulation between miR-222-3p and CALM1. Silencing miR-222-3p (inhibitor) upregulated CALM1 mRNA levels, and simultaneously upregulated AQP5 mRNA 1.8-fold, whereas silencing CALM1 (siCALM1) increased miR-222-3p expression (*P* < 0.05, Fig. 3C). This mutual repression suggests a feedback loop wherein CALM1 stabilizes TUG1 by sequestering miR-222-3p. Critically, TUG1 overexpression elevated CALM1 expression, an effect reversed by miR-222-3p mimic. Silencing CALM1 abolished the anti-inflammatory and antioxidant effects of TUG1, restoring LPS-induced TNF-α, IL-6, and IL-1β levels (mRNA: Fig. 3D; ELISA: Fig. 3E). Although variability was relatively high (SD ≈ 12%), the regulatory trends remained highly significant (*P* < 0.01), and exacerbating oxidative stress (Fig. 3F). A schematic model illustrates the ceRNA mechanism^24^: TUG1 competitively binds miR-222-3p, alleviating its repression of CALM1 mRNA and enabling CALM1 translation (Fig. 3G). These findings unequivocally establish TUG1 as a ceRNA that safeguards CALM1 expression by sponging miR-222-3p.

### TUG1 activates the CALM1/PRKG1/RYR3/AQP5 pathway through miR-222-3p suppression

**Fig. 4:**
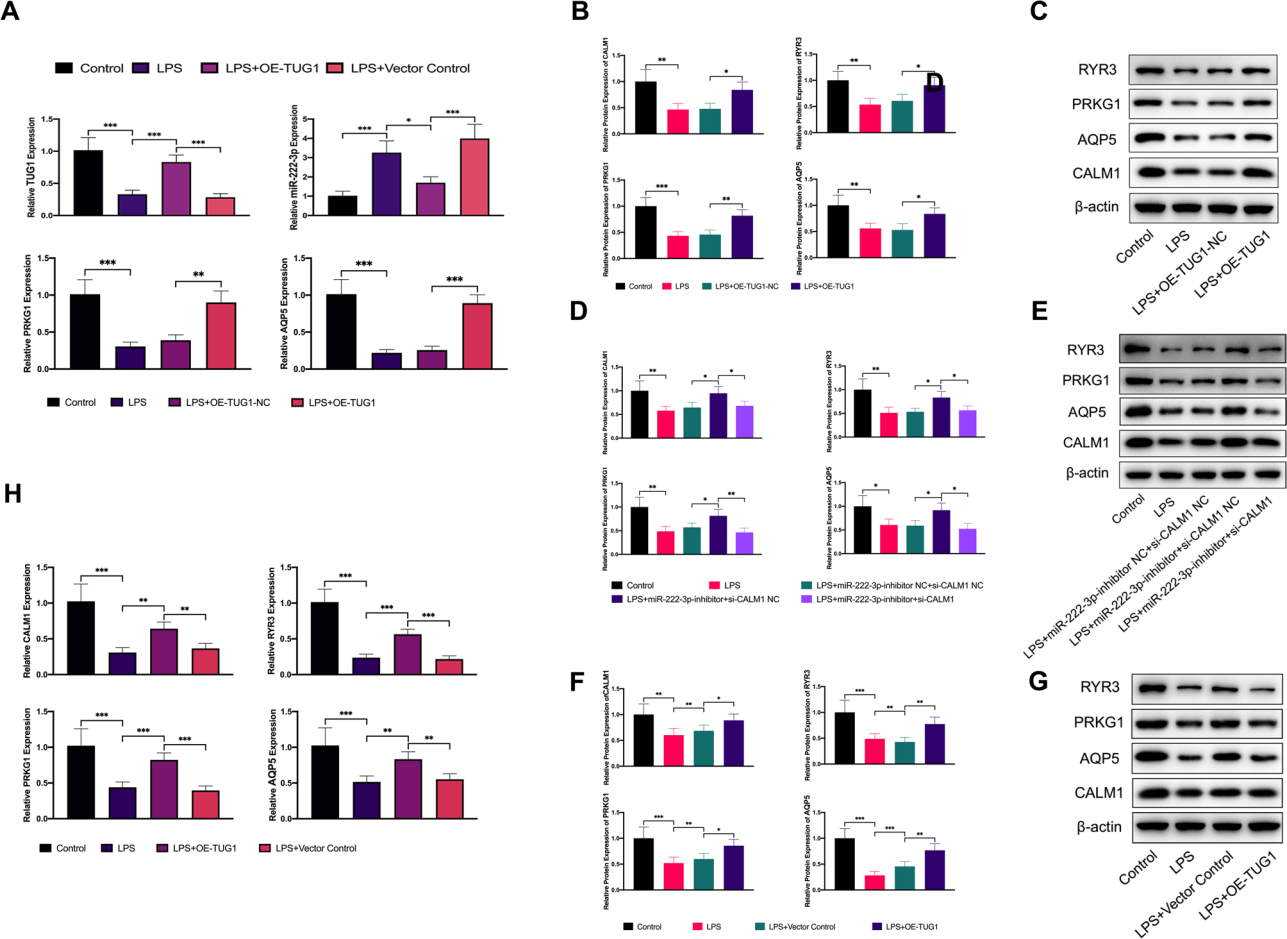

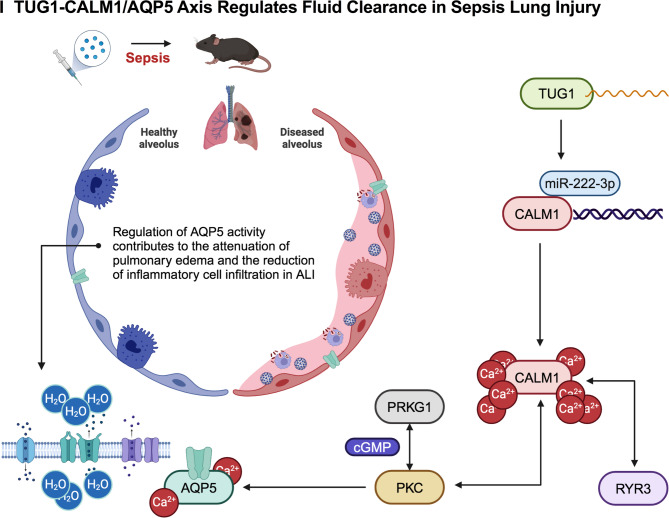
TUG1 activates the CALM1/PRKG1/RYR3/AQP5 axis by suppressing miR-222-3p **(A)** qPCR analysis of CALM1, PRKG1, RYR3, and AQP5 mRNA levels in LPS-treated MLE-12 cells transfected with OE-TUG1 or vector control (OE-NC). **(B)** Quantitative bar graphs of CALM1, PRKG1, RYR3, and AQP5 protein expression determined by western blot (WB). **(C)** Representative WB images corresponding to panel B. *Data: mean ± SD, *n* = 3; *P < 0.05 vs. LPS + OE-NC (one-way ANOVA). **(D)** WB analysis of CALM1, PRKG1, RYR3, and AQP5 following miR-222-3p inhibition, with or without CALM1 knockdown. **(E)** Representative WB bands for panel D. *Data: mean ± SD, *n* = 3; *P < 0.05 vs. anti-NC; #*P* < 0.05 vs. anti-miR-222-3p + siNC. **(F)** WB quantification of CALM1, PRKG1, RYR3, and AQP5 in lung tissue samples. **(G)** Corresponding WB images. **(H)** qPCR of CALM1, PRKG1, RYR3, and AQP5 mRNA in lung tissues. *Data: mean ± SD, *n* = 5; *P < 0.05 vs. LPS + AAV-NC (Student’s t-test). **(I)** Mechanistic diagram: TUG1 sequesters miR-222-3p, relieving its inhibition of CALM1, which in turn activates the PRKG1/RYR3 signaling pathway and upregulates AQP5 to promote alveolar fluid clearance. LPS impairs this cascade by downregulating TUG1 and upregulating miR-222-3p. The schematic illustration in panel (I) was created with BioRender. Original uncropped blots are presented in the Supplementary Material.

To delineate the downstream signaling cascade regulated by TUG1, we examined the expression of CALM1, PRKG1, RYR3, and AQP5 in LPS-treated MLE-12 cells. LPS significantly downregulated mRNA and protein levels of all four genes, while TUG1 overexpression restored their expression (*P* < 0.05, Fig. 4A). Notably, AQP5, a critical regulator of alveolar fluid clearance, exhibited the most pronounced rescue effect, with TUG1 overexpression boosting AQP5 protein levels by 2.1-fold (Fig. 4B-C, *P* < 0.01), supporting enhanced alveolar fluid clearance. To validate the dependency of this pathway on the TUG1/miR-222-3p/CALM1 axis, we silenced CALM1 in miR-222-3p-inhibited cells. Silencing miR-222-3p alone upregulated CALM1, PRKG1, RYR3, and AQP5 protein levels, but concurrent CALM1 knockdown resulted in an approximately 80% reversal of these protective effects (*P* < 0.05, Fig. 4D-E), confirming CALM1 as the central node. Consistent with in vitro findings, LPS-induced ALI mice showed reduced CALM1/PRKG1/RYR3/AQP5 expression in lung tissues, which was rescued by TUG1 overexpression at both mRNA and protein levels (*P* < 0.05, Fig. 4F-H). A schematic model integrates these findings: TUG1 sequesters miR-222-3p to derepress CALM1, which activates PRKG1/RYR3-dependent signaling, ultimately upregulating AQP5 to enhance alveolar fluid transport via its hydrophobic pore domain (Fig. 4I). These results demonstrate that TUG1 orchestrates the CALM1/PRKG1/RYR3/AQP5 axis to counteract LPS-induced alveolar dysfunction.

*The data are presented as mean ± standard deviation (SD), *n* = 3. Statistical significance was assessed using one-way ANOVA. *P < 0.05, **P < 0.01, **P < 0.001 compared to the control group.

### TUG1 overexpression attenuates LPS-induced ALI in vivo

**Fig. 5:**
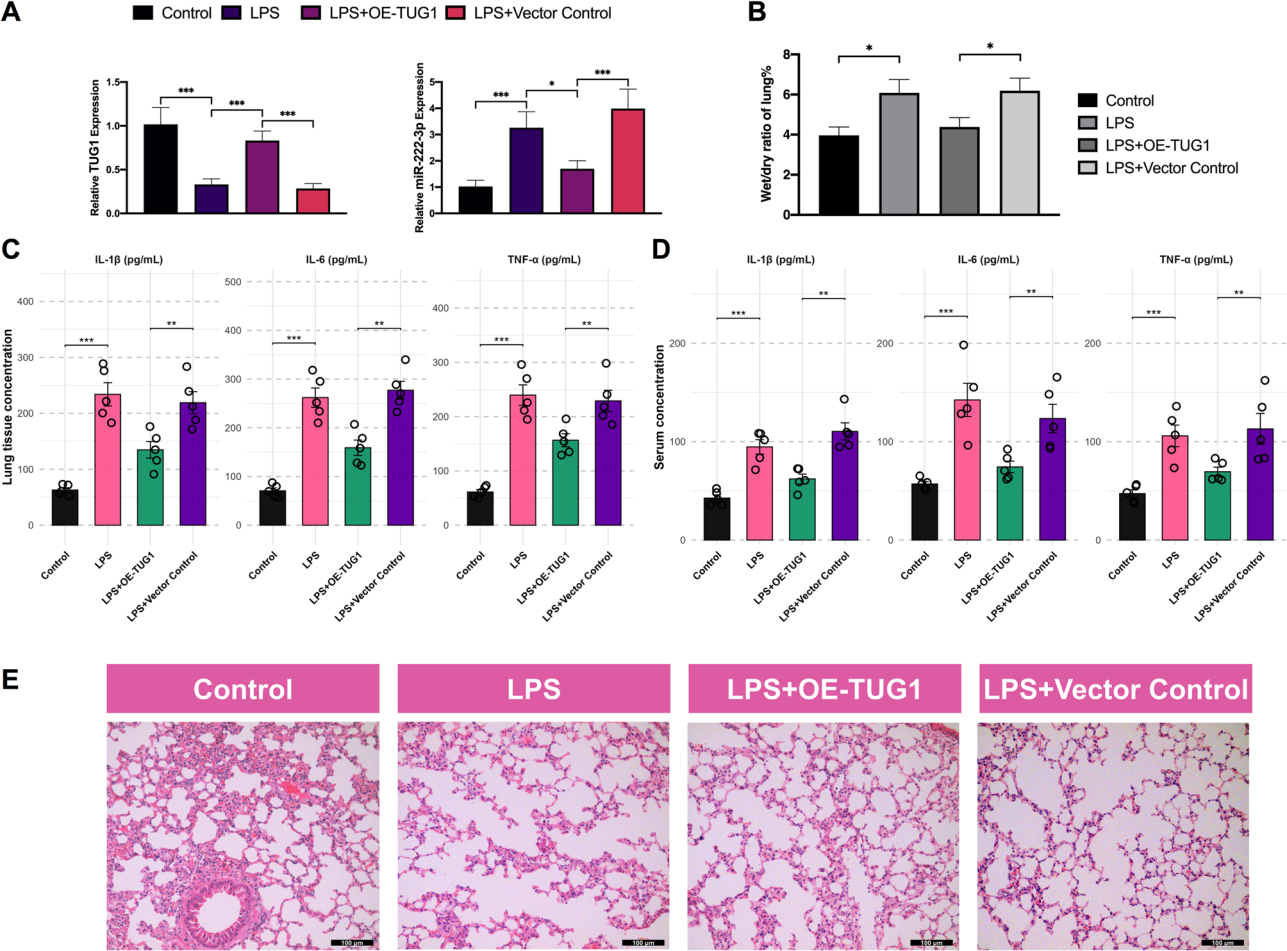
TUG1 mitigates LPS-induced ALI in mice. **(A)** The expression levels of TUG1 and miR-222-3p in mouse lung tissues were measured using quantitative PCR (qPCR). **(B)** The lung wet/dry weight ratio was determined to assess the degree of pulmonary edema, a hallmark of ALI. A significant reduction in the wet/dry weight ratio in the TUG1 overexpression group (LPS + OE-TUG1) compared to the LPS-treated group indicates that TUG1 may attenuate the extent of lung edema induced by LPS. **(C-D)** The levels of pro-inflammatory cytokines TNF-α, IL-6, and IL-1β were assessed in lung homogenates and serum using ELISA. Increased levels of these cytokines were observed in the LPS-treated group, indicating a robust inflammatory response associated with ALI. However, TUG1 overexpression significantly reduced the secretion of these inflammatory mediators both in the lungs and in serum, further supporting the anti-inflammatory role of TUG1 in this model. **(E)** Representative histological images of lung tissue sections stained with Hematoxylin and Eosin (H&E) (scale bar: 100 μm) demonstrate the structural changes in the lung. *The results are expressed as mean ± standard deviation (SD), with n = 5 for each group. Statistical analysis was performed using one-way ANOVA. Values of *P < 0.05, **P < 0.01, and ***P < 0.001 were considered statistically significant.

To validate the therapeutic role of TUG1 in vivo, we established an LPS-induced ALI mouse model. TUG1 expression was significantly downregulated in LPS-treated lung tissues, whereas intratracheal delivery of TUG1 overexpression vectors restored its levels. Conversely, miR-222-3p was upregulated 2.3-fold by LPS (Fig. 5A, *P < 0.001, consistent with GSE datasets) and suppressed by TUG1, consistent with in vitro findings. LPS challenge increased lung wet/dry weight ratio (LPS group 1.85 ± 0.12 vs. Control), indicating severe pulmonary edema. However, TUG1 overexpression reduced this ratio (OE-TUG1 1.45 ± 0.09, P < 0.01, Cohen’s d = 1.5; Fig. 5B). Histopathological analysis (H&E staining) revealed distinct morphological changes across groups: Control group: Normal lung architecture with intact alveoli, thin alveolar walls, and minimal inflammatory infiltration. LPS group: Severe alveolar damage, characterized by collapsed alveoli, thickened septa, intra-alveolar hemorrhage, and dense inflammatory cell infiltration. LPS + OE-TUG1 group: Attenuated pathological changes, with preserved alveolar structure, reduced edema, and fewer inflammatory cells. Blinded inflammation scoring on a 0–4 scale quantitatively supported this, showing a significant decrease from 3.2 in the LPS group to 1.8 in the OE-TUG1 group(Fig. 5E). Consistent with histopathology, TUG1 overexpression suppressed LPS-induced TNF-α, IL-6, and IL-1β levels in both lung tissues P < 0.05, Fig. 5C) and systemic circulation (P < 0.05, Fig. 5D). These findings confirm that TUG1 alleviates LPS-induced ALI in vivo by mitigating pulmonary edema, inflammation, and histopathological damage.

### Validation of the TUG1-related regulatory axis using public sepsis transcriptomic datasets

To establish the clinical relevance of the TUG1-mediated signaling axis identified in our experimental models, we examined two publicly available sepsis-related transcriptomic datasets (GSE241238 and GSE48080).

#### GSE241238 analysis (PBMCs from septic patients vs. healthy controls)

**Fig. 6:**
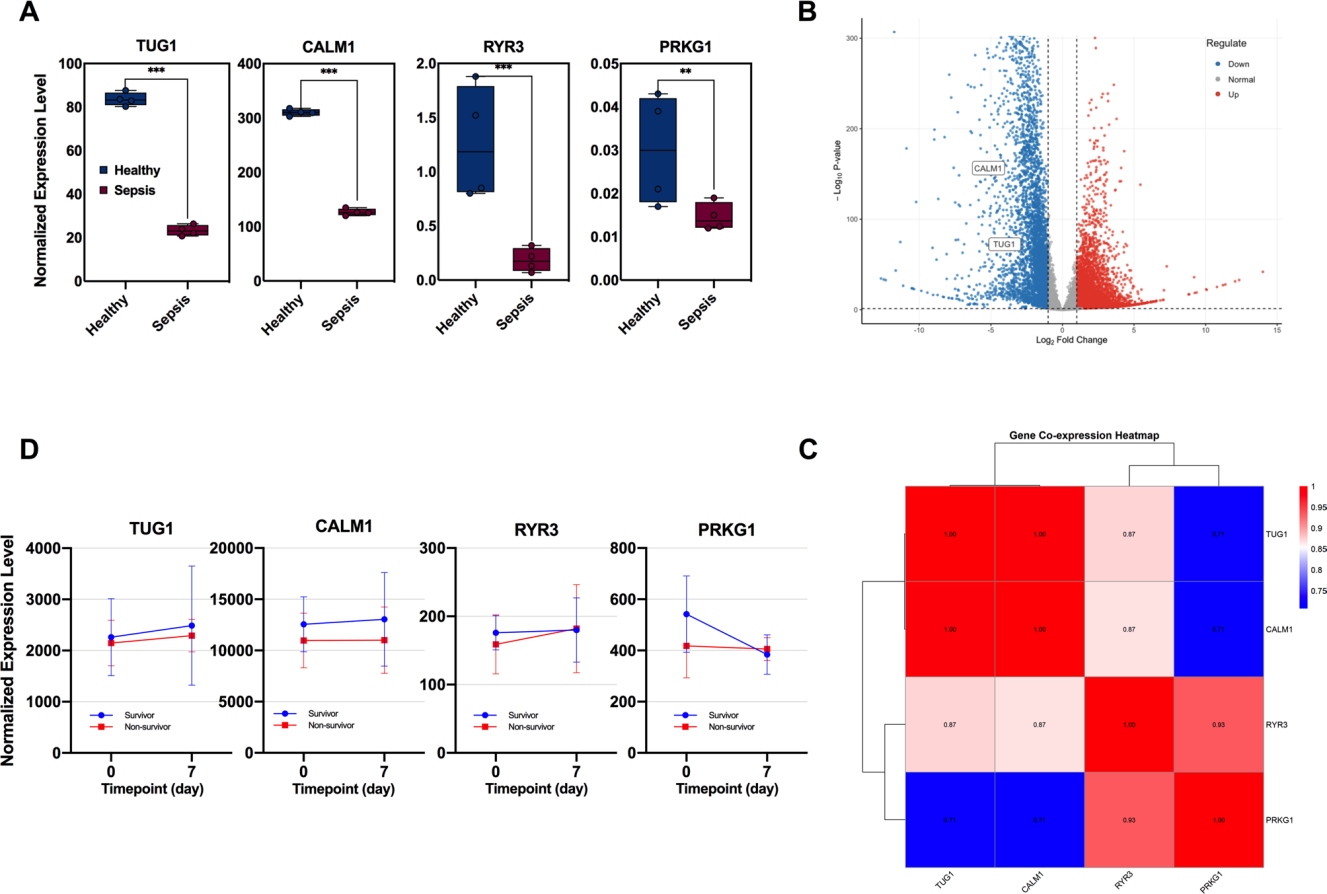
Clinical validation of the TUG1-mediated regulatory axis in sepsis. **(A)** Boxplots illustrating significantly reduced expression levels of TUG1, CALM1, PRKG1, and RYR3 in peripheral blood mononuclear cells (PBMCs) from septic patients compared to healthy controls (GSE241238). **(B)** Volcano plot of transcriptomic changes in sepsis, with TUG1 and CALM1 highlighted as significantly downregulated transcripts (log₂FC < − 1.5, *P* < 0.01). **(C)** Co-expression heatmap (Pearson correlation coefficients) revealing strong positive correlations between TUG1 and CALM1 (*r* = 0.995), as well as coordinated expression between CALM1, PRKG1, and RYR3. **(D)** Longitudinal expression patterns of TUG1, CALM1, and RYR3 at Day 0 and Day 7 in survivors versus non-survivors of pneumonia-associated sepsis (GSE48080). Expression levels are presented as mean ± SD in arbitrary units (AU), derived from normalized transcriptomic signal intensities. *P* < 0.05 by Student’s t-test. *Correspondence to: Dr. Lyu, Email: iculvliwen@163.com.

In line with observations from our LPS-induced injury models, the expression levels of TUG1, CALM1, RYR3, and PRKG1 were significantly decreased in peripheral blood mononuclear cells (PBMCs) from septic patients compared to healthy individuals (*P* < 0.05; Fig. 6A). Differential expression analysis revealed TUG1 and CALM1 among the most significantly downregulated genes (log2FC < − 1.5, *P* < 0.01; Fig. 6B). Pearson correlation analysis demonstrated a strong positive association between TUG1 and CALM1 expression (*r* = 0.995, 95% CI 0.98–0.99, *P* < 0.001), indicating strong co-expression, as well as substantial correlations between CALM1 and PRKG1 (*r* = 0.714), and PRKG1 and RYR3 (*r* = 0.933, *P* < 0.001; Fig. 6C). These findings are consistent with our mechanistic model, which proposes that TUG1 stabilizes CALM1 expression to promote downstream RYR3/PRKG1 signaling.

#### GSE48080 analysis (survivors vs. non-survivors in pneumonia-associated sepsis)

Baseline expression of TUG1 and CALM1 was significantly higher in survivors compared to non-survivors (TUG1: 2263 vs. 2148 AU; CALM1: 12,552 vs. 10,966 AU; *P* < 0.05; Fig. 6D–E). Over the 7-day observation period, survivors exhibited progressive upregulation of TUG1 (+ 9.9%) and CALM1 (+ 4.0%), while non-survivors showed a 0.4-fold TUG1 drop at Day 7 (*P* < 0.05), linked to poor prognosis (Fig. 6D). Expression of RYR3 also remained relatively stable in survivors (176 to 180 AU), in contrast to a more variable pattern in non-survivors (159 to 182 AU; *P* > 0.05; Fig. 6F). Although PRKG1 expression transiently declined in survivors (Day 7: 384 vs. 542 AU), its strong correlation with RYR3 (*r* = 0.933) in the GSE241238 dataset supports a functionally relevant interaction within this axis.

#### Clinical-pathological implications

The consistent downregulation of TUG1, CALM1, RYR3, and PRKG1 in human sepsis—coupled with their selective restoration in survivors—underscores their putative roles as critical regulators of inflammation resolution. Notably, the exceptionally high co-expression between TUG1 and CALM1 (*r* = 0.995) reinforces our experimental findings that TUG1 promotes CALM1 expression by sequestering miR-222-3p. Although AQP5 was not included in these datasets, we strictly acknowledge that these clinical transcriptomic data are derived from PBMCs and whole blood, which primarily capture the systemic inflammatory immune landscape and disease severity of sepsis, rather than the local alveolar epithelial microenvironment. Therefore, while Fig. 6 robustly underscores the clinical prognostic value and systemic relevance of the TUG1/CALM1 axis, the direct functional evidence supporting its specific role in AQP5-mediated alveolar fluid clearance is exclusively derived from our in vitro MLE-12 and in vivo murine lung models.

## Discussion

Sepsis is a severe, infection-driven condition characterized by dysregulated systemic inflammation, frequently leading to acute lung injury (ALI), which manifests as alveolar-capillary barrier dysfunction, excessive neutrophil infiltration, and fluid accumulation in the lungs^25,26^. The lungs are particularly susceptible to septic insult due to their rich vascularization and continuous exposure to circulating pathogens. Despite improvements in critical care, the mortality rate of ALI remains over 40%, largely due to persistent hypoxemia and fluid overload^27,28^. This highlights the ongoing need to better understand the molecular regulators of alveolar repair. In recent years, long non-coding RNAs (lncRNAs) have emerged as critical modulators of sepsis pathophysiology^29^. For example, MALAT1 promotes ALI by sponging miR-149 and enhancing MyD88-dependent inflammation^30^. TUG1, a lncRNA primarily recognized for its tumor-suppressive roles^31,32^, has shown context-dependent effects in sepsis—attenuating renal and intestinal injury via the miR-200c-3p/SIRT1 and miR-34b-5p/GAB1 axes^33],]^ but paradoxically aggravating macrophage-mediated inflammation.

This study suggests the TUG1 axis protects against ALI, though larger cohorts are needed for extensive validation.Our in vitro, in vivo, and clinical dataset analyses suggest that downregulation of TUG1 during septic ALI may lead to derepression of miR-222-3p, which in turn suppresses CALM1 expression. As a critical calcium sensor, CALM1 coordinates PRKG1/RYR3 signaling, which regulates AQP5 activation and alveolar fluid clearance. Disruption of this axis appears to exacerbate pulmonary inflammation and edema in our model. Dataset analyses (GSE241238, GSE48080) are consistent with our findings, as they show higher expression of TUG1 and CALM1 in survivors of pneumonia-associated sepsis, consistent with a protective role in disease resolution. Furthermore, Fig. 6D and dataset trends show persistent miR-222-3p elevation in non-survivors, suggesting a poor prognosis (future Kaplan-Meier [KM] survival validation is required).

The ceRNA regulatory model has gained prominence in understanding sepsis-induced organ dysfunction, particularly in modulating inflammation and repair at the post-transcriptional level^34^. Our results suggest the existence of a lung-specific ceRNA network in which TUG1 may act as a sponge for miR-222-3p, a microRNA previously linked to calcium homeostasis^35^. Dual-luciferase assays validated direct interactions among TUG1, miR-222-3p, and the CALM1 3′UTR. In LPS-treated alveolar epithelial cells, TUG1 deficiency led to miR-222-3p upregulation and CALM1 suppression, disrupting calcium signaling and epithelial barrier integrity^36,37^. At the inflammatory level, TUG1 reduces cytokine mRNA expression, potentially via NF-κB inhibition (though future ChIP validation is needed to confirm direct transcriptional regulation).Compared with TUG1’s functions in renal or cardiac injury^38,39^, this lung-specific mechanism underscores its regulatory plasticity. Moreover, the strong correlation between TUG1 and CALM1 in public datasets suggests evolutionary conservation and therapeutic relevance. These results complement previous findings on MALAT1 and suggest that TUG1 may function as a dual regulator of inflammation and fluid transport in alveolar epithelial cells.

The CALM1-mediated calcium signaling cascade is a key regulator of ALI resolution, coordinating both anti-inflammatory signaling and alveolar fluid clearance^40^. Based on our findings and prior mechanistic insights, we propose a putative regulatory axis involving CALM1, PRKG1, RYR3, and AQP5 in the context of sepsis-induced lung injury. Although direct interactions among these molecules have not been comprehensively defined, existing evidence supports plausible functional links. CALM1, upon calcium binding, is known to activate downstream kinases and modulate calcium channels^41,42^. PRKG1, a calcium-sensitive kinase, can be indirectly influenced by CALM1 activity and has been implicated in epithelial barrier function and AQP regulation. RYR3, a ryanodine receptor responsible for intracellular calcium release, may be stabilized by PRKG1 or CALM1-mediated signaling^42^, thereby sustaining calcium flux necessary for epithelial responses. Sustained intracellular calcium, in turn, facilitates AQP5 trafficking to the membrane, promoting fluid clearance^43^. While PRKG1 may enhance AQP5 function via PKC-dependent phosphorylation^44,45^, RYR3 ensures proper localization through calcium microdomain formation^46^. Our results indicate that TUG1 overexpression restores CALM1 expression and may partially reactivate this calcium-dependent pathway, ultimately reducing alveolar edema. Although the structural and direct biochemical interactions between these molecules remain to be fully elucidated, to our knowledge, this is the first study to propose a functional link among these components within a unified axis relevant to ALI. Furthermore, while AQP5 was not directly detected in public transcriptomic datasets, the Fig. 6C heatmap shows strong co-expression of CALM1/PRKG1/RYR3 (*r* > 0.90), indirectly supporting AQP5 recovery and its potential involvement is supported by our experimental observations and previous reports linking AQP5 to ARDS severity^47^.

### Limitations and Future Directions

Several limitations warrant consideration. First, this study primarily utilized TUG1 overexpression models; complementary loss-of-function strategies (e.g., siRNA knockdown or CRISPR-Cas9 editing) are necessary to confirm its essential regulatory role in ALI. Furthermore, while our dual-reporter assays support the proposed ceRNA network, future Ago2-RIP assays are required to confirm direct endogenous interactions. Importantly, the downstream physiological functions of the PRKG1/RYR3/AQP5 axis—such as real-time calcium flux (e.g., via Fluo-4/AM imaging), precise aquaporin-mediated fluid transport (e.g., via TEER assays), and specific downstream phosphorylation events (e.g., p-PRKG1)—were not quantified in the current study. These functional assays represent critical next steps to establish strict causality rather than indirect associations. Second, the specific cellular contributors to this axis—particularly epithelial versus immune compartments—remain undefined, warranting the development of lineage-specific knockout models. Additionally, a critical limitation regarding our clinical translational data is that the public datasets (GSE241238, GSE48080) utilized PBMCs. Consequently, these findings reflect circulating immune responses and serve as systemic biomarkers of the TUG1/CALM1 axis in sepsis, rather than providing direct human epithelial evidence for alveolar fluid clearance. Third, although our data suggest a sequential functional relationship among CALM1, PRKG1, RYR3, and AQP5, the direct molecular mechanisms underpinning this cascade remain incompletely understood. For instance, whether CALM1 modulates PRKG1 activity directly, or indirectly influences RYR3-dependent calcium flux, is yet to be established. Similarly, the precise coordination between PRKG1-mediated phosphorylation and RYR3-mediated membrane trafficking in regulating AQP5 function remains speculative. Further studies employing high-resolution approaches—such as cryo-electron microscopy, Förster resonance energy transfer (FRET), or co-immunoprecipitation assays—will be essential to clarify the spatial and structural dynamics of this pathway^48–50^. Finally, future research should focus on systematically dissecting this TUG1–CALM1–PRKG1/RYR3–AQP5 axis under inflammatory stress conditions, particularly in clinically relevant models such as aged or comorbid animals. Clarifying the validity and therapeutic relevance of this signaling cascade could inform the development of new strategies to enhance alveolar fluid clearance in sepsis-induced lung injury. Collectively, while our study provides a basis for the proposed TUG1–CALM1–PRKG1/RYR3–AQP5 axis, the current evidence remains preliminary, and further validation is essential to confirm its mechanistic and therapeutic relevance.

## Conclusion

This study identifies a novel lncRNA-mediated axis—TUG1/miR-222-3p/CALM1—that promotes alveolar repair in sepsis-induced ALI by coordinating anti-inflammatory signaling and fluid clearance. TUG1 restoration activates a calcium signaling cascade involving CALM1, PRKG1, RYR3, and AQP5, enhancing alveolar fluid resorption. Our findings represent the first integration of these genes into a unified pathway in the context of sepsis, highlighting TUG1 as a potential therapeutic target for ALI.

## Supplementary Information

Below is the link to the electronic supplementary material.

Supplementary material 1 (PDF 422.0 kb)

Supplementary material 2 (PDF 1125.8 kb)

## Data Availability

The datasets analyzed in this study were downloaded from the publicly available Gene Expression Omnibus (GEO) database. All experimental data generated during this study are available from the corresponding author upon reasonable request.
