# Supplementary material for: LncRNA TUG1 mitigates sepsis-induced acute lung injury via a ceRNA network regulating the CALM1/PRKG1/RYR3/AQP5 axis
Source: Sci Rep. 2026 May 16;16:22295. doi: 10.1038/s41598-026-51003-1 (PMC13376352; doi:10.1038/s41598-026-51003-1)
Supplement: Supplementary file 1 — Supplementary material 1 (PDF 422.0 kb) [file 41598_2026_51003_MOESM1_ESM.pdf]

广西壮族自治区人民医院伦理委员会批件

编号：KY-KJT-2023-005 号

广西壮族自治区人民医院李喆：

本伦理委员会于 2023 年 7 月 25 日审查你们的项目“lncRNA TUG1/miR-222-3p/CALM1 轴在脓毒症肺损伤中的作用及机制研究（主动健康与常见病多发病联合专项）”，同意你们自该日起开展该项研究。上述资料未经本伦理委员会批准，不得有任何改动。

主任委员或副主任委员：林全富

2023 年 7 月 25 日

广西壮族自治区人民医院
伦理委员会
4501000264270

地址：广西南宁市桃源路 6 号　　邮编：530021

联系人：黎洪棉、莫晓云　　联系电话：0771-2186214　　传真：0771-2186214
