# Supplementary material for: LncRNA TUG1 mitigates sepsis-induced acute lung injury via a ceRNA network regulating the CALM1/PRKG1/RYR3/AQP5 axis
Source: Sci Rep. 2026 May 16;16:22295. doi: 10.1038/s41598-026-51003-1 (PMC13376352; doi:10.1038/s41598-026-51003-1)
Supplement: Supplementary file 2 — Supplementary material 2 (PDF 1125.8 kb) [file 41598_2026_51003_MOESM2_ESM.pdf]

Supplementary Material

LncRNA TUG1 mitigates sepsis-induced acute lung injury via a ceRNA network regulating the CALM1/PRKG1/RYR3/AQP5 axis

Zhe Li, Wan Chen, Lei Shi, Guozheng Qiu, Yao zhou, Yanlin Wei, Zhengzhuang Huang, Liwen Lyu*

Original Uncropped Western Blots for Figure 4

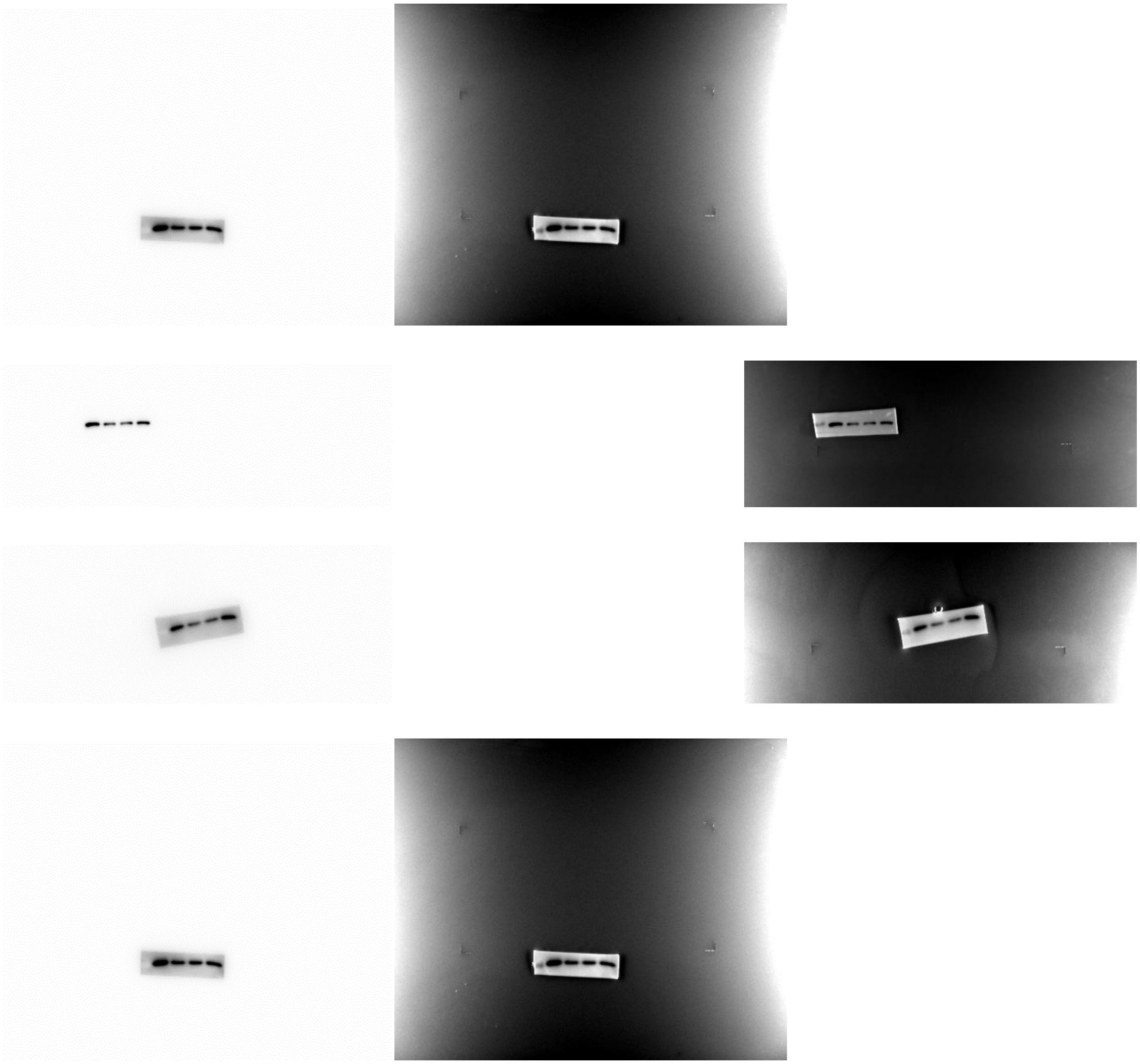

Supplementary Figure S1. Original uncropped blot for AQP5. This blot corresponds to the AQP5 panel shown in Figure 4C of the main manuscript.

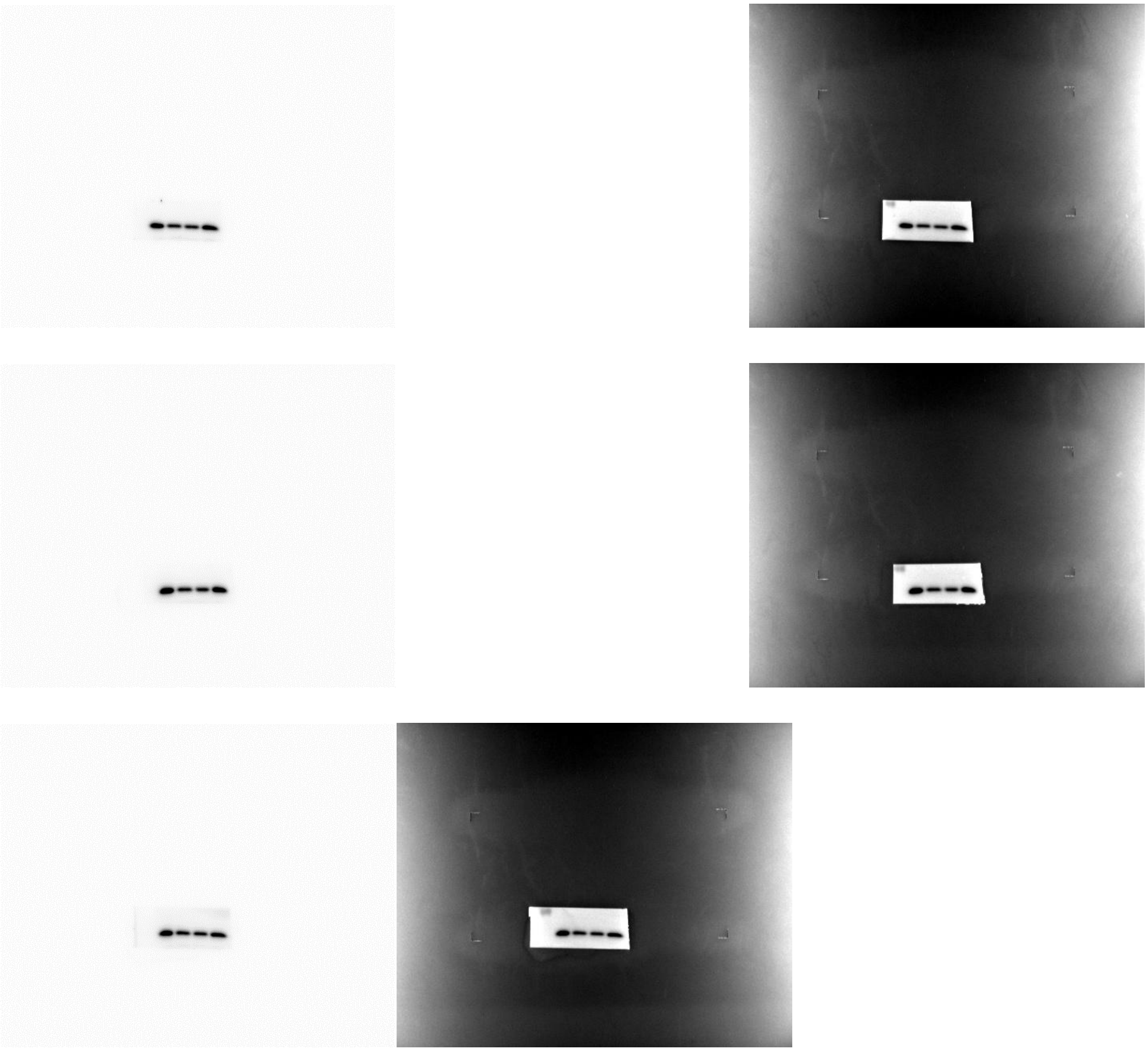

Supplementary Figure S2. Original uncropped blot for CALM1. This blot corresponds to the CALM1 panel shown in Figure 4C of the main manuscript.

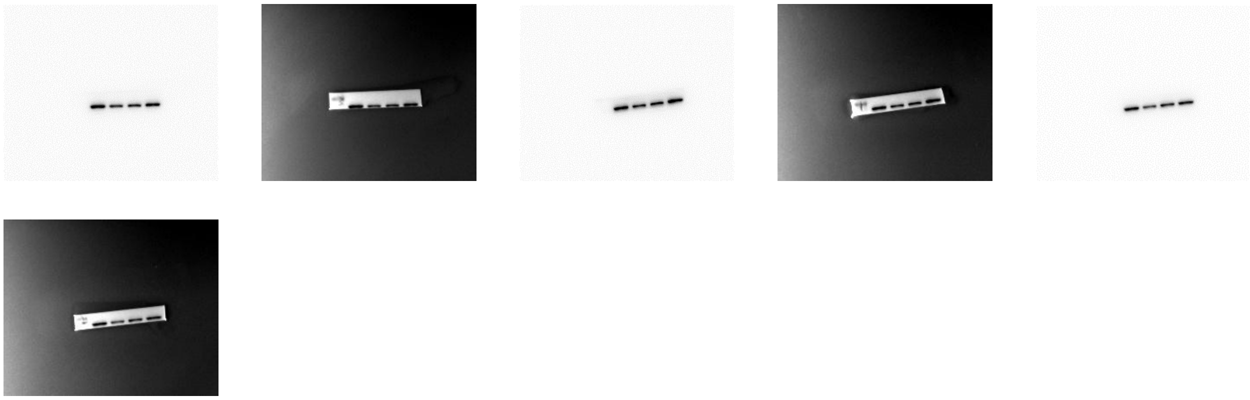

Supplementary Figure S3. Original uncropped blot for PRKG1. This blot corresponds to the PRKG1 panel shown in Figure 4C of the main manuscript.

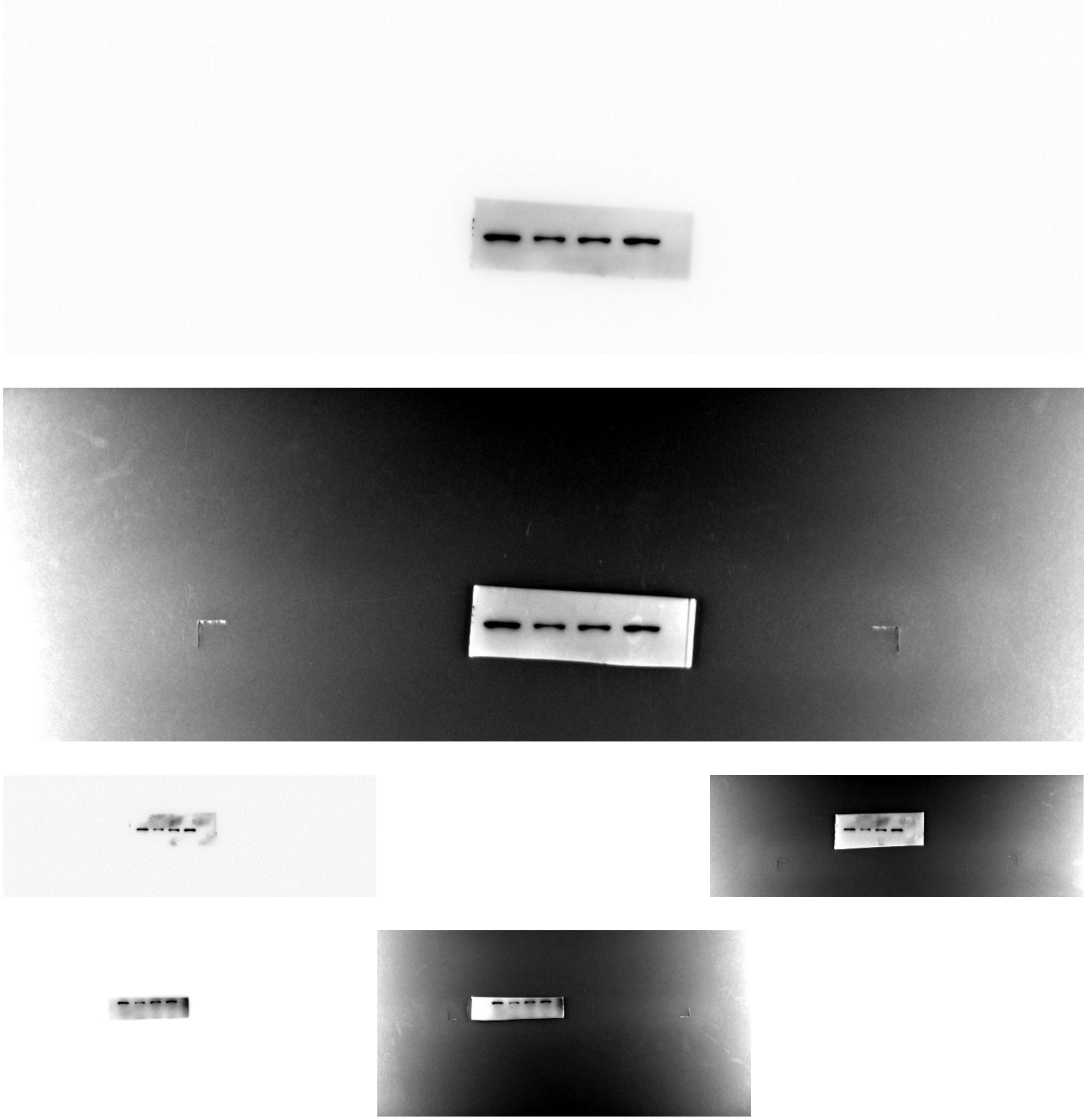

Supplementary Figure S4. Original uncropped blot for RYR3. This blot corresponds to the RYR3 panel shown in Figure 4C of the main manuscript.

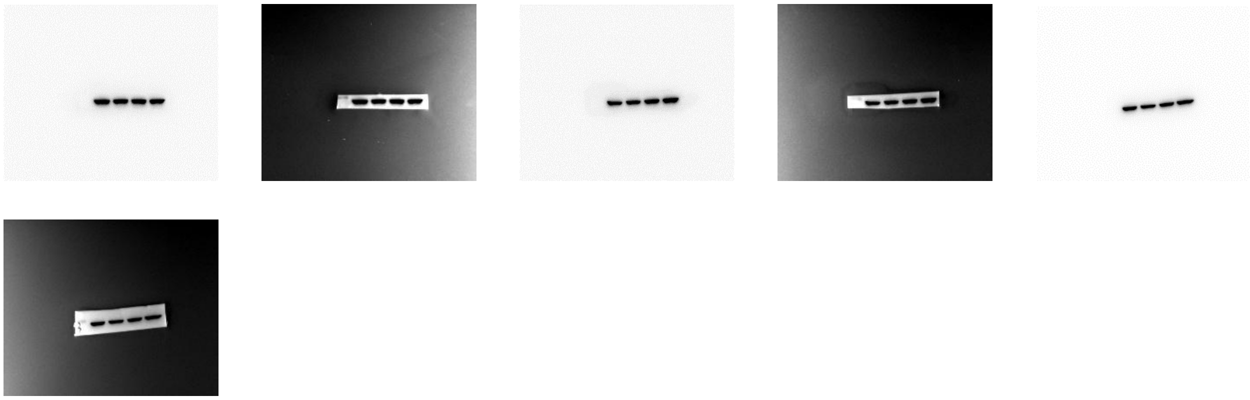

Supplementary Figure S5. Original uncropped blot for β-actin. This blot corresponds to the **β-**actin panel shown in Figure 4C of the main manuscript.

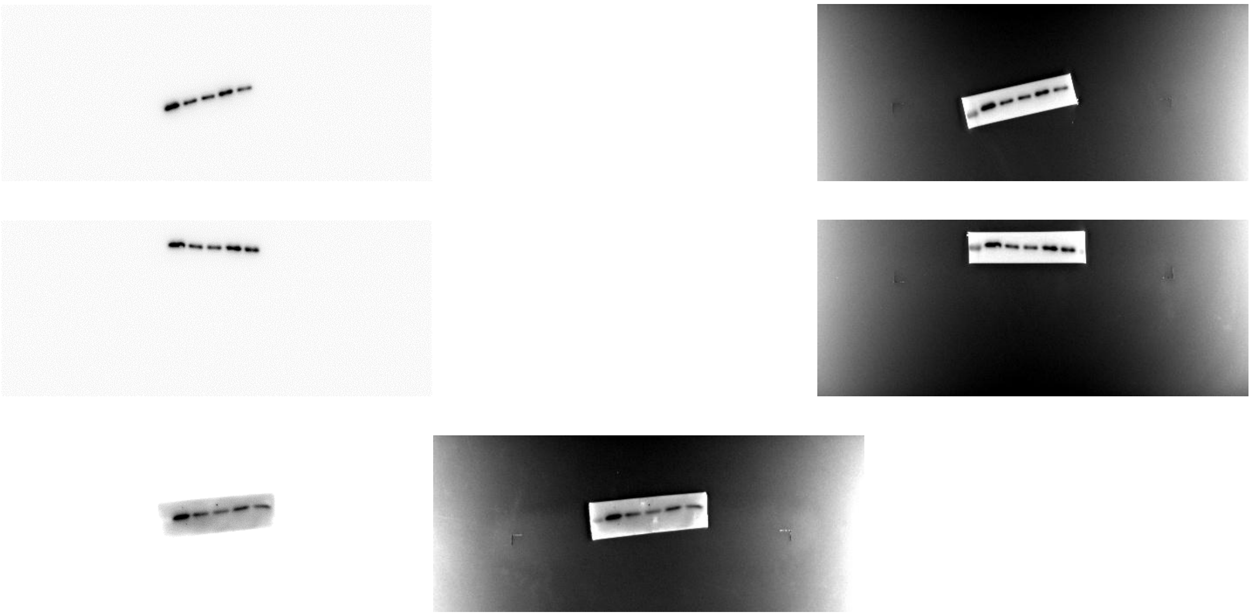

Supplementary Figure S6. Original uncropped blot for AQP5. This blot corresponds to the AQP5 panel shown in Figure 4E of the main manuscript.

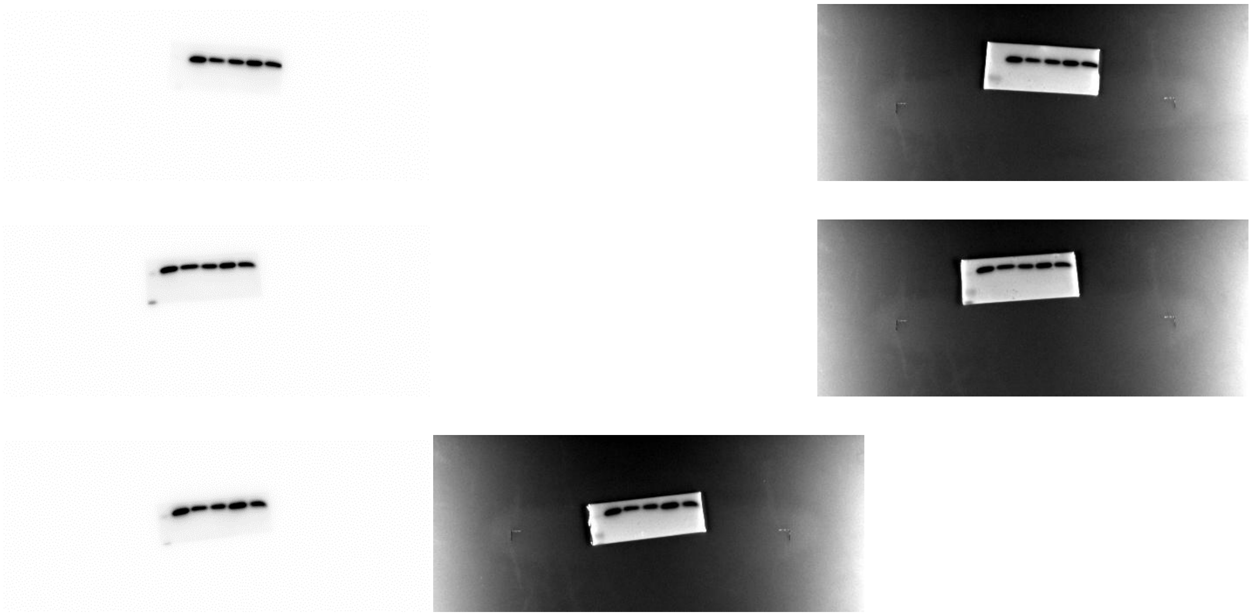

Supplementary Figure S7. Original uncropped blot for CALM1. This blot corresponds to the CALM1 panel shown in Figure 4E of the main manuscript.

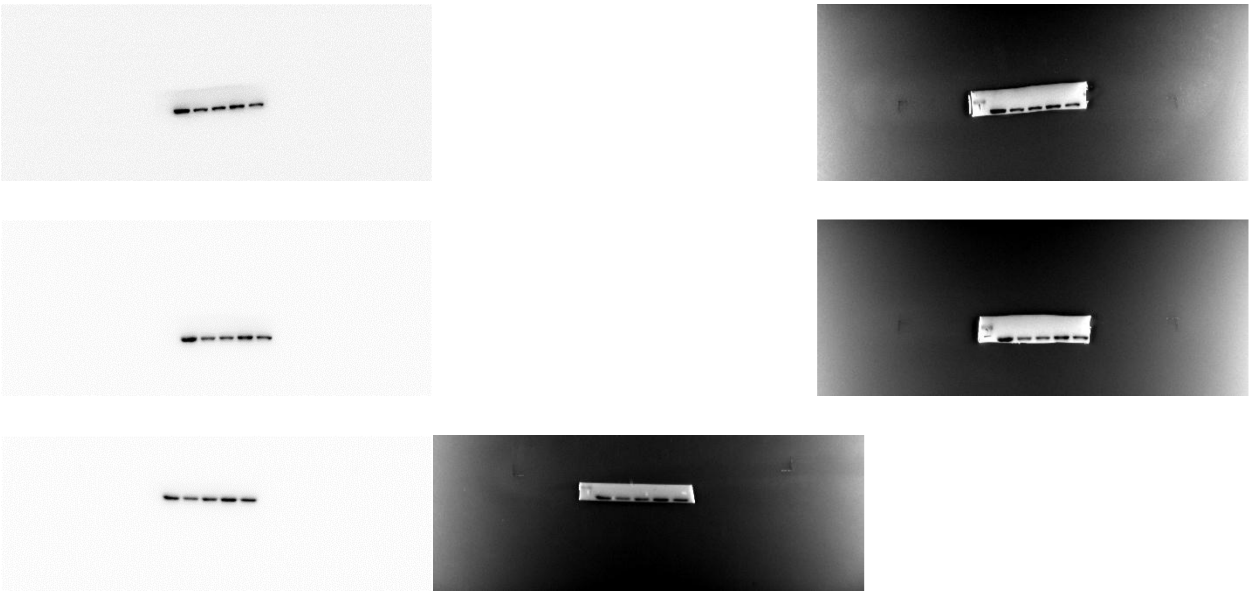

Supplementary Figure S8. Original uncropped blot for PRKG1. This blot corresponds to the PRKG1 panel shown in Figure 4E of the main manuscript.

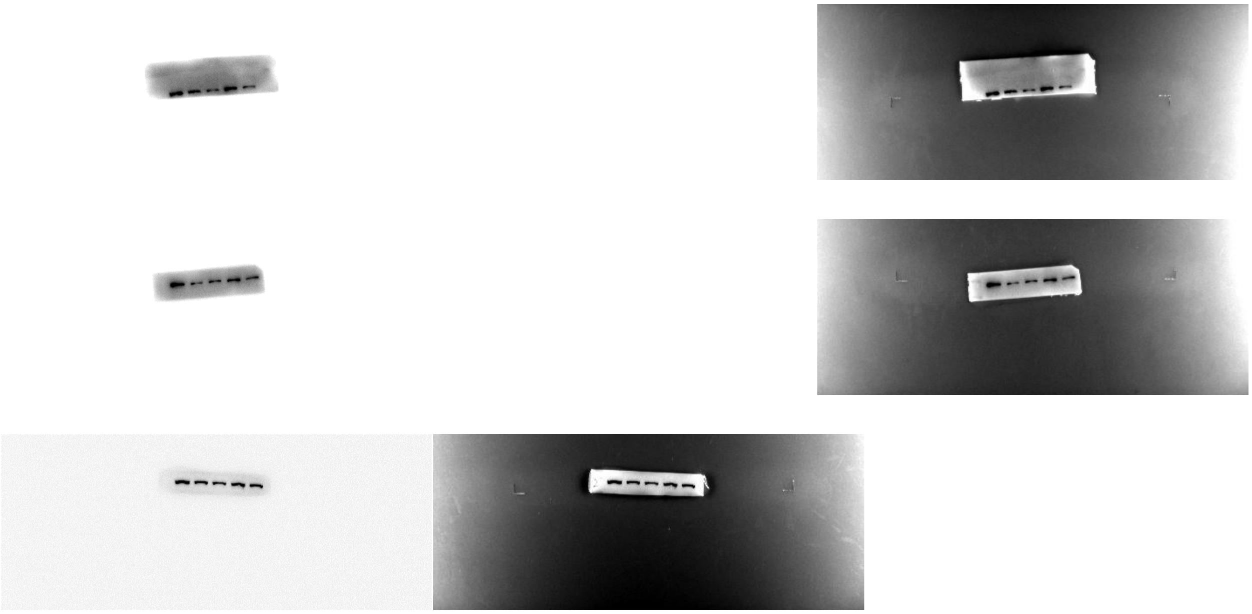

Supplementary Figure S9. Original uncropped blot for RYR3. This blot corresponds to the RYR3 panel shown in Figure 4E of the main manuscript.

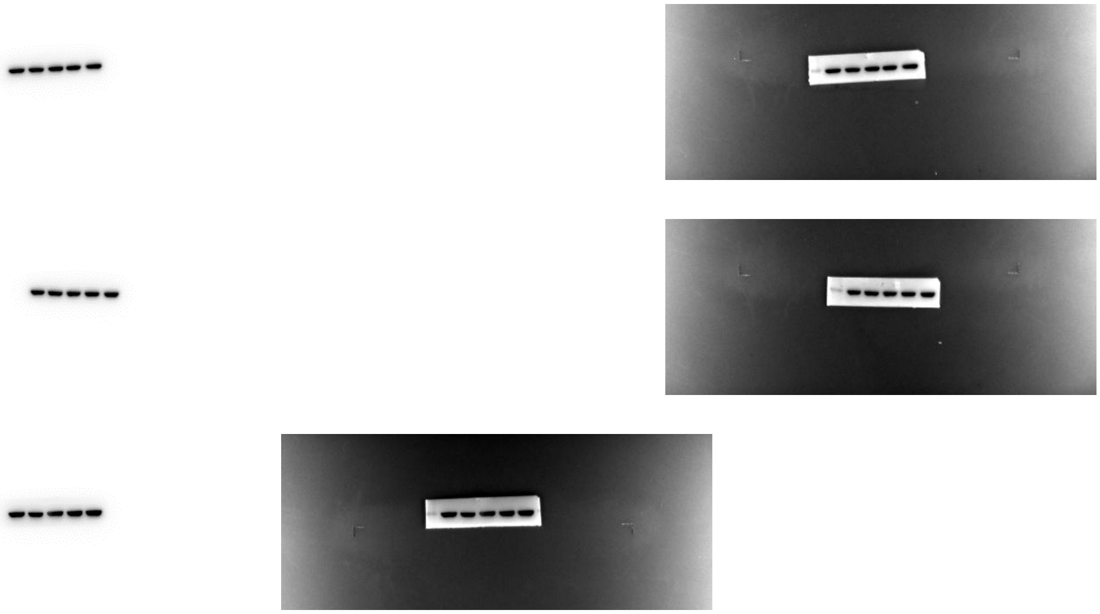

Supplementary Figure S10. Original uncropped blot for β-actin. This blot corresponds to the β-actin panel shown in Figure 4E of the main manuscript.

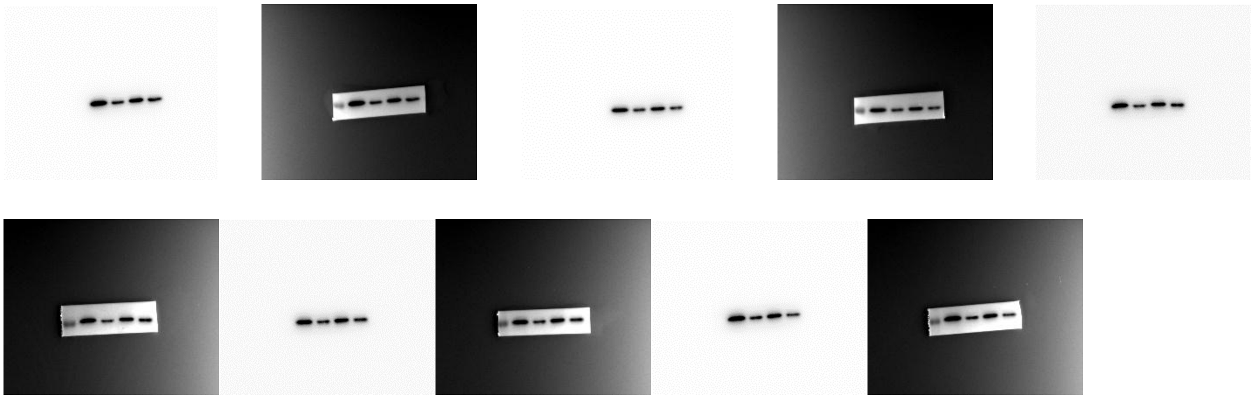

Supplementary Figure S11. Original uncropped blot for AQP5. This blot corresponds to the AQP5 panel shown in Figure 4G of the main manuscript.

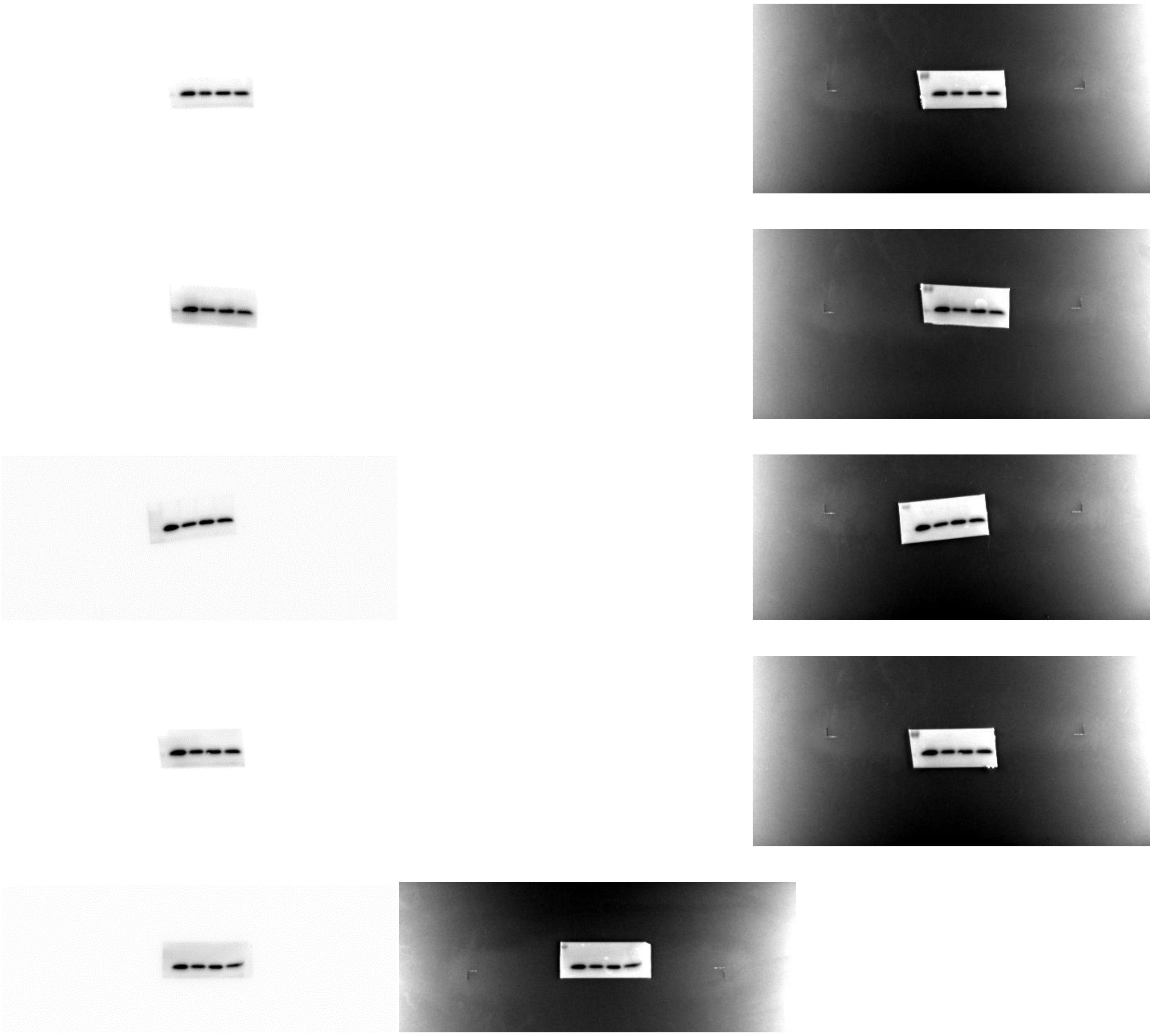

Supplementary Figure S12. Original uncropped blot for CALM1. This blot corresponds to the CALM1 panel shown in Figure 4G of the main manuscript.

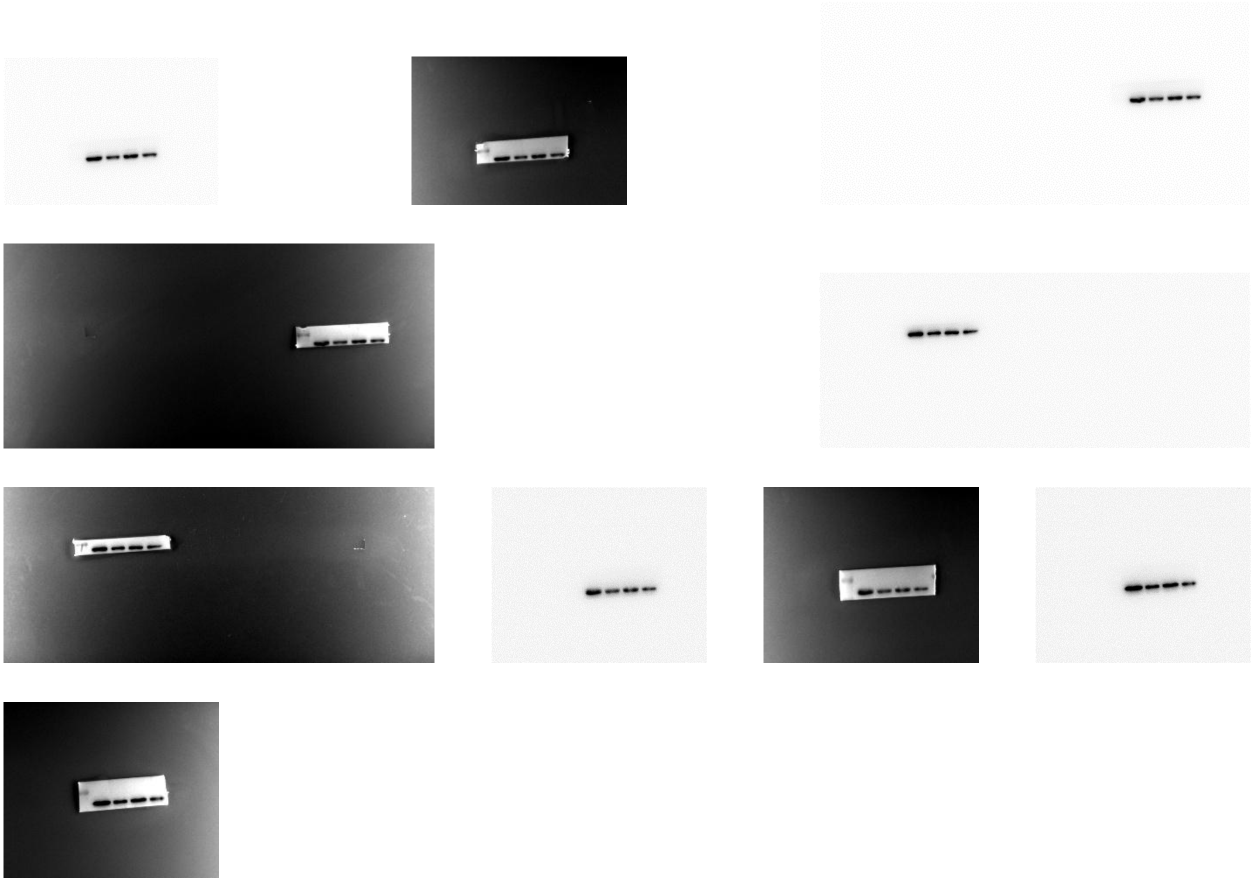

Supplementary Figure S13. Original uncropped blot for PRKG1. This blot corresponds to the PRKG1 panel shown in Figure 4G of the main manuscript.

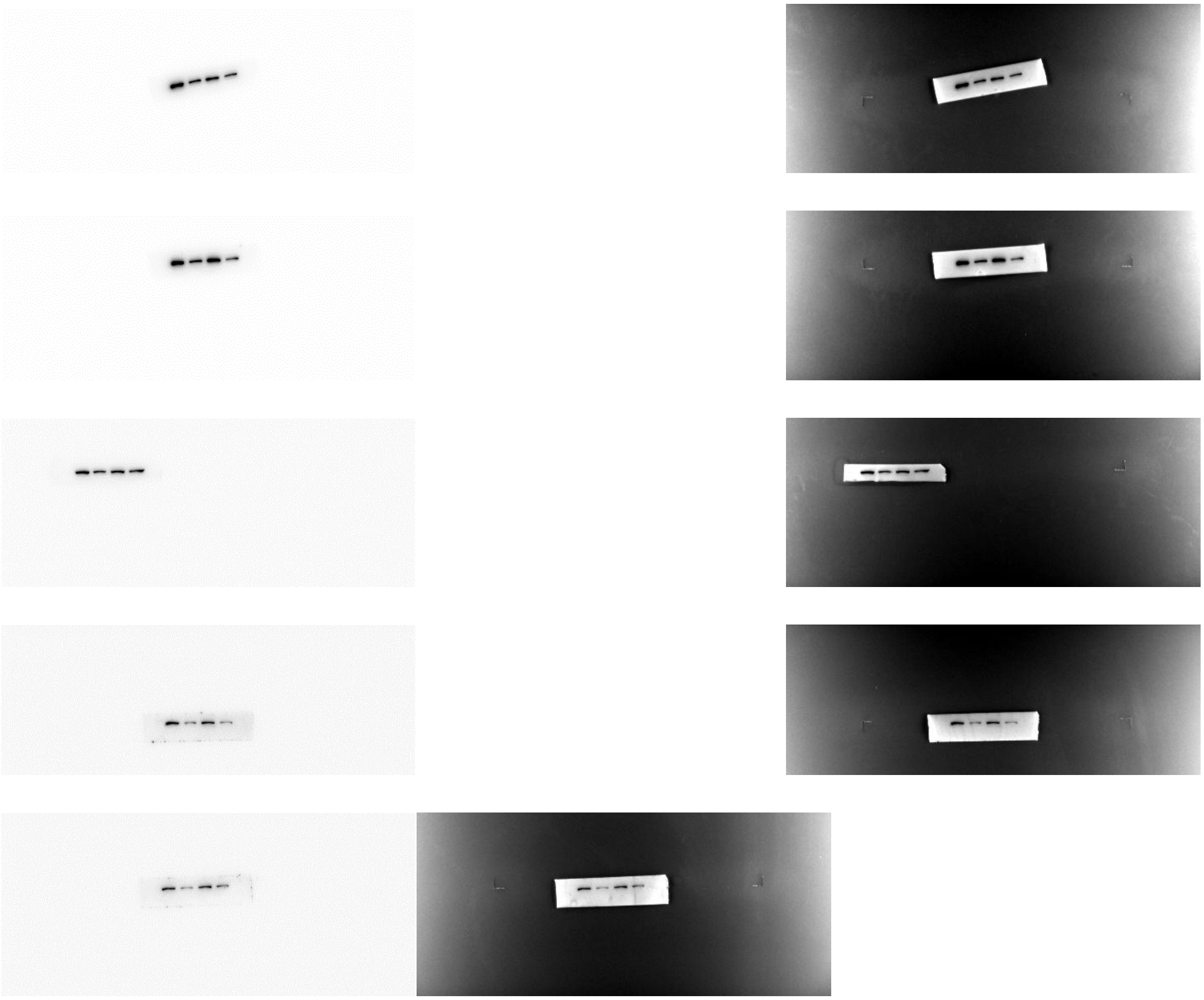

Supplementary Figure S14. Original uncropped blot for RYR3. This blot corresponds to the RYR3 panel shown in Figure 4G of the main manuscript.

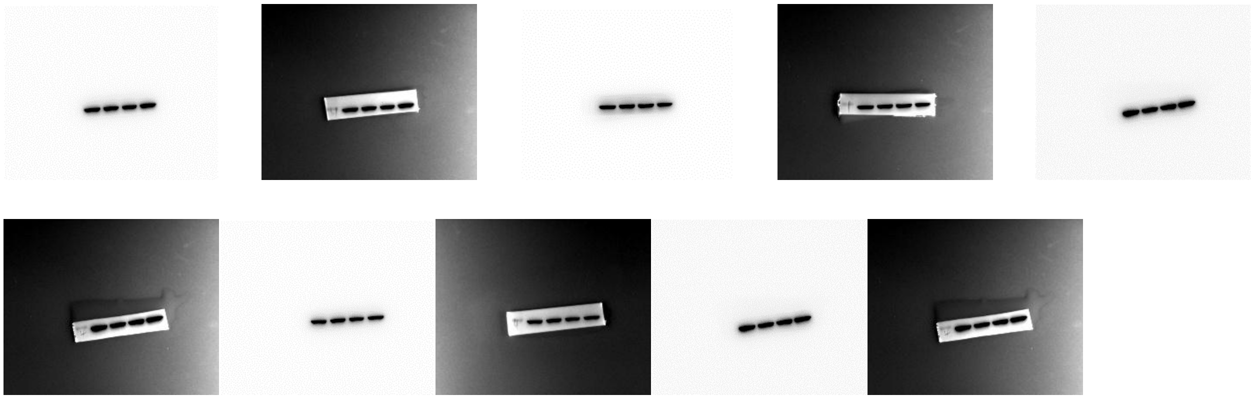

Supplementary Figure S15. Original uncropped blot for β-actin. This blot corresponds to the β-actinpanel shown in Figure 4G of the main manuscript.
